# Comorbidity and Multimorbidity in Adults With Congenital Heart Disease: Findings From a Multi‐Site Population‐Based Study

**DOI:** 10.1002/bdr2.2515

**Published:** 2025-08-21

**Authors:** Lorenzo D. Botto, Matthew R. Reeder, George K. Lui, M. Jill Glidewell, Wendy M. Book, Tessa L. Crume, Jesse M. DeLaRosa, Alfred d'Ottavio, Karrie F. Downing, Marcia L. Feldkamp, Daphne T. Hsu, Amber D. Khanna, Sergey Krikov, Nelangi M. Pinto, Cheryl L. Raskind Hood, Fred H. Rodriguez, Aida S. Soim, Kevin J. Whitehead, Karen Chiswell, Jennifer S. Li

**Affiliations:** ^1^ Department of Pediatrics University of Utah Salt Lake City Utah USA; ^2^ Stanford University School of Medicine Palo Alto California USA; ^3^ National Center on Birth Defects and Developmental Disabilities, Centers for Disease Control and Prevention Atlanta Georgia USA; ^4^ Division of Cardiology Emory University School of Medicine Atlanta Georgia USA; ^5^ Department of Epidemiology Emory University Rollins School of Public Health Atlanta Georgia USA; ^6^ Colorado School of Public Health University of Colorado Anschutz Medical Center Aurora Colorado USA; ^7^ Duke Clinical Research Institute Durham North Carolina USA; ^8^ Children's Hospital at Montefiore and Albert Einstein College of Medicine Bronx New York USA; ^9^ Division of Cardiology, Departments of Internal Medicine and Pediatrics, Colorado School of Medicine University of Colorado Anschutz Medical Campus Aurora Colorado USA; ^10^ Division of Cardiology, Department of Pediatrics University of Washington Seattle Washington USA; ^11^ New York State Department of Health Albany New York USA; ^12^ Division of Cardiovascular Medicine, Department of Internal Medicine University of Utah Salt Lake City Utah USA; ^13^ Department of Pediatrics Duke University Medical Center Durham North Carolina USA

**Keywords:** adults, comorbidity, disparity, epidemiology, heart defects congenital, multimorbidity

## Abstract

**Background:**

Survival of individuals with congenital heart disease (CHD) has improved, leading to a growing and aging population of adults living with these conditions. Over their lifetime, they often face an array of comorbidities that affect outcomes and complicate medical management. However, population‐based information on such comorbidities is scarce, reducing opportunities for prevention.

**Methods:**

This population‐based, cross‐sectional study assessed comorbid conditions in adults with CHD residing in five geographic areas in the United States (in Colorado, Georgia, New York, North Carolina, and Utah). The study included 18,672 adults aged 19 to 64 years who had a healthcare encounter between 2011 and 2013 associated with ≥ 1 CHD‐related diagnosis code. Data were derived from linked clinical and administrative sources, reflecting inpatient, outpatient, and emergency department encounters.

**Results:**

Most adults with CHD experienced at least one (88.5%) and usually multiple (76%) comorbidities. Overall, noncardiac comorbidities exceeded cardiac comorbidities. The most frequent noncardiac comorbidities were endocrine/metabolic conditions (e.g., diabetes, hyperlipidemia, hypothyroidism), hypertension, and neuropsychiatric conditions (e.g., anxiety, depression). The presence and number of comorbidities varied in different sociodemographic groups. Men and older individuals experienced higher rates of many comorbidities, cardiac and noncardiac, regardless of CHD type.

**Conclusions:**

Preventable and treatable comorbidity and multimorbidity are common in adults with CHD, with patterns shaped by sociodemographic factors and CHD type. Reducing preventable mortality in this growing population will require sustained tracking of health metrics and coordinated, data‐driven, and lifelong care.

**Abbreviations**
CCI: Charlson comorbidity index
CCS: clinical classifications software
FQE: first qualifying encounter

## 1 Introduction

Congenital heart disease (CHD), which occurs in at least 1 in 100 newborns, is transitioning from being predominantly a neonatal and pediatric condition to a complex lifelong challenge for an increasing number of adults (Liu et al. 2023; Diller et al. 2021). The prevalence of adults with CHD in North America is estimated at approximately 1 in every 150 adults, amounting to about 1.4 million adults in the United States (US) alone (Gilboa et al. 2016; Marelli et al. 2014). In Canada, adults with CHD outnumber children by a two‐to‐one ratio (Marelli et al. 2014; Gilboa et al. 2016; Warnes et al. 2001). With advancements in medical and surgical care, similar demographic shifts are expected globally.

Over their lifespan, individuals with CHD are at risk for comorbidities that complicate clinical management and increase the risk of adverse health outcomes (Lui et al. 2017; Bracher et al. 2017; Oster et al. 2021; Neidenbach et al. 2018; Maurer et al. 2021; Liu et al. 2023; Agarwal et al. 2019; Billett et al. 2008), leading some researchers to note that the disease burden in CHD patients is shifting “away from the heart and toward acquired cardiovascular and systemic complications” (Liu et al. 2023). This shift risks increasing the “healthspan gap”—the disparity between increased life expectancy and the extension of healthspan—driven by the rising prevalence of chronic conditions in an aging population (Liu et al. 2023; Garmany et al. 2021; Lui et al. 2017).

Despite its critical importance for clinical care and healthcare planning, the current understanding of comorbid and multimorbid conditions in individuals with CHD remains incomplete, especially on a population‐based level (Forman et al. 2018; Lui et al. 2017; Liu et al. 2023). Existing data often originates from specific communities that may not represent the general population, such as those available through specific referral centers, hospital systems, or health insurance networks (Lui et al. 2017; Bracher et al. 2017; Neidenbach et al. 2018; Agarwal et al. 2019; Konstantinos Dimopoulos et al. 2008; Yang et al. 2020).

To address these gaps and better understand the impact of CHD across the lifespan, the Centers for Disease Control and Prevention (CDC), in partnership with local public health and academic institutions, initiated the Lifespan study in five geographic areas across the US (Jill Glidewell et al. 2021; Glidewell et al. 2018). This report presents findings from the Lifespan study focusing on comorbidity and multimorbidity in adults with CHD.

## 2 Materials and Methods

### 2.1 Study Design and Population

The Lifespan study is a retrospective cohort study of adolescents and adults with CHD residing at any time from 1 January 2011 to 31 December 2013 in one of five geographic areas of the US: Colorado, North Carolina, Utah, five counties in metropolitan Atlanta (Georgia), and 11 counties in the state of New York (see *supplemental file* for the list of counties) (Glidewell et al. 2018; Jill Glidewell et al. 2021). Their comorbidities were documented in the same three‐year surveillance period (2011–2013).

This report from the Lifespan study focuses on adults aged 19 to 64. Age was defined as the individual's age at the first qualifying encounter (FQE) during this period. FQE was defined as the first encounter between 2011 and 2013 with an eligible CHD code (listed below). Our methods to identify CHD cases via multiple linked administrative and clinical databases have been described in detail (Glidewell et al. 2018; Jill Glidewell et al. 2021) and briefly summarized here.

### 2.2 Data Sources

For Colorado, statewide cases were identified using electronic health records (EHR) from four independent healthcare systems that serve diverse populations across the state and the Colorado All Payer Claims Database. For North Carolina, statewide cases were identified from EHR data from five pediatric and adult comprehensive CHD clinical facilities. For Utah, statewide cases were identified using hospital discharge data and clinical data from the state's two major healthcare systems (University of Utah and Intermountain Health) with pediatric and adult cardiology specialty clinics. For Georgia (selected counties), cases were identified using Medicaid claims data, data from six pediatric and adult clinical facilities, and an existing surgical database. For New York State (specifically, selected counties), cases were identified through three pediatric cardiology clinics, Medicaid claims data, and hospital inpatient and outpatient discharge data. Each site determined vital status by linking to state death certificates and retained this information in the surveillance dataset. Individuals who died during the surveillance period were not excluded from the surveillance system. Cases were linked and de‐duplicated across participating case‐finding data sources at each site.

### 2.3 Inclusions and Exclusions

Included individuals had a recorded health encounter with a qualifying *International Classification of Diseases, Ninth Revision, Clinical Modification* (ICD‐9‐CM) CHD code. Recorded health encounters include those in clinical and administrative databases of inpatient admissions, emergency department visits, and outpatient ambulatory visits. Qualifying ICD‐9‐CM codes included 745.××–747.××, with the following exclusions: 745.5 (atrial septal defect vs. patent foramen ovale), 746.86 (congenital heart block); 747.32 (pulmonary arteriovenous malformation/aneurysm); 747.5× (hypoplasia of umbilical artery); 747.6× (other anomalies of peripheral vascular system); 747.8× (other anomalies of circulatory system). Individuals with one of the exclusion codes above (e.g., for congenital heart block) who also had a qualifying code (e.g., for tetralogy of Fallot) were included in the study.

### 2.4 Cardiac Severity Groups

Individuals were classified into one of five mutually exclusive CHD severity groups: severe, shunt, valve, shunt‐valve (i.e., both types of CHD present), and “other”. This analysis included individuals with severe, shunt‐valve, valve, and shunt conditions. The severity groups were based on a previously described method that categorizes CHD diagnostic codes into mutually exclusive hierarchical groups based on cardiac anatomy (Glidewell et al. 2018), similarly to prior studies (Marelli et al. 2014). The *supplemental file* provides details of the assignment algorithm. Severe CHD included endocardial cushion defects, interrupted aortic arch, tetralogy of Fallot, total anomalous pulmonary venous return, tricuspid atresia, transposition complexes, truncus arteriosus, and univentricular hearts. Individuals with multiple CHD‐related ICD‐9‐CM codes who had at least one severe code were classified as having a severe condition regardless of the number of non‐severe codes they had.

### 2.5 Comorbidities

We used the Charlson comorbidity index as a broad summary of comorbidity (Charlson et al. 1987, 2022). The Charlson score was designed as an assessment tool to predict long‐term mortality based on 19 conditions (Charlson et al. 1987, 2022). We used the unweighted version of this index as a descriptor of the overall cohort and selected subgroups (e.g., by CHD type). ICD‐9‐CM diagnoses were categorized into comorbidity categories using the Clinical Classifications Software (CCS) system, developed by the US Agency for Healthcare Research and Quality (AHRQ 2023), to capture specific conditions clinically relevant to individuals with CHD. Categories were then modified and condensed into 26 groups based on literature review and expert clinician input from the study team. We also removed ICD‐9‐CM codes representing symptoms or signs (e.g., chest pain) rather than a comorbidity (see *supplemental file* for details).

### 2.6 Covariates

In addition to the CHD groups, the main covariates in the analyses included age group (19–24, 25–34, 35–44, 45–54, 55–64), sex (male, female), race (multiracial, white, black, other, unknown), ethnicity (Hispanic, non‐Hispanic, unknown), study site (Atlanta, Colorado, New York, North Carolina, Utah), and insurance type (public, private, none, unknown). If an individual had documented Medicaid or Medicare insurance at any time during 2011–2013, they were classified as having public insurance. Private insurance was defined as any documented private, other government (including Tricare), or other insurance, but no indication of receipt of public insurance during 2011–2013. Individuals with documented self‐pay or uninsured were classified as having no insurance (i.e., none), and unknown was defined as unavailable or unknown.

### 2.7 Analysis

We compared the distribution of the main covariates across the main CHD severity groups using the Kruskal‐Wallis rank sum test for median age and Pearson's chi‐squared test for all other categorical variables; statistics are provided with false discovery rate correction for multiple testing. We calculated the number of adults with and without comorbidities, overall and by each covariate. In addition to Kruskal‐Wallis rank‐sum and Pearson's chi‐squared tests, we used multivariable regression models to examine the associations between comorbidities as the outcome variable and covariates as independent variables. Comorbidity was evaluated as a dichotomous variable (at least one vs. no comorbidities) and as a continuous (number of comorbidities). We used Poisson regression models with robust error variance to obtain estimates of incidence rate ratios (IRR) and conservative 95% confidence intervals (CI). We used negative binomial regression models in the presence of overdispersion (variance larger than mean), as was the case in the distribution of the number of comorbidities.

For covariates with > 1% missing values, we repeated these analyses using multiple imputation techniques (see *supplemental file* for missing data analysis), including multiple imputation by chained equations (MICE). MICE is a robust statistical technique that uses multivariate models to iteratively predict the missing variables based on the observed data (without the missing data). This process generates multiple complete datasets, allowing for more accurate effect estimation by addressing the uncertainty associated with missing data (Buuren and Groothuis‐Oudshoorn 2011; Schafer 1999).

We evaluated the frequency of major comorbidity patterns (e.g., at least one comorbidity, multimorbidity, non‐cardiac comorbidity, specific types of comorbidities) overall and by sex, CHD severity group, race/ethnicity, and age group. Differences in proportions between two groups were tested with a *t*‐test of equality of proportions. Statistical analyses were done separately using the SAS software package and R (version 4.3.2, 2023‐10‐31), 2001–2023 R Core Team, using RStudio (2023.12.1 Build 402, 2009–2024 Posit Software, PBC) and were replicated by an independent team. The R code and output were compiled in a Quarto document to enhance reproducibility.

## 3 Results

After exclusions based on age and presence of ineligible CHD codes (Figure S1), the final study cohort included 18,672 adults with CHD (severe, shunt, valve, shunt‐valve) who were 19 to 64 years old between 2011 and 2013 and had a documented CHD‐coded healthcare encounter during the same period.

### 3.1 Cohort Characteristics

Table 1 describes the cohort by sociodemographic characteristics and CHD severity group. Severe CHD accounted for 21% of cases. Valve lesions were the largest CHD group, accounting for 52% of cases.

**TABLE 1:** Selected characteristics of adults (19–64 years old) with congenital heart disease (CHD), overall and by CHD group, Lifespan study, 2011–201.

Characteristic	*N*	Overall, *N* = 18,672	Congenital heart disease group				*p* ^a^	*q*‐value^b^
			Severe, *N* = 3911 (21%)	Shunt‐valve, *N* = 930 (5.0%)	Shunt, *N* = 4197 (22%)	Valve, *N* = 9634 (52%)		
Age, median (IQR)	18,672	38 (27–52)	30 (24–40)	33 (25–47)	36 (26–50)	45 (30–55)	< 0.001	< 0.001
Age group, *n* (%)	18,672						< 0.001	< 0.001
19–24		3628 (19)	1112 (28)	225 (24)	871 (21)	1420 (15)		
25–34		4449 (24)	1387 (35)	263 (28)	1121 (27)	1678 (17)		
35–44		3303 (18)	718 (18)	172 (18)	770 (18)	1643 (17)		
45–54		3629 (19)	420 (11)	149 (16)	764 (18)	2296 (24)		
55–64		3663 (20)	274 (7.0)	121 (13)	671 (16)	2597 (27)		
Sex, *n* (%)	18,668						< 0.001	< 0.001
Female		9665 (52)	2137 (55)	549 (59)	2780 (66)	4199 (44)		
Male		9003 (48)	1774 (45)	381 (41)	1415 (34)	5433 (56)		
Race, *n* (%)	15,215						< 0.001	< 0.001
White		12,019 (79)	2548 (77)	635 (81)	2170 (68)	6666 (84)		
Black		2331 (15)	570 (17)	98 (13)	734 (23)	929 (12)		
Other		506 (3.3)	112 (3.4)	28 (3.6)	183 (5.7)	183 (2.3)		
Multiracial		359 (2.4)	89 (2.7)	20 (2.6)	101 (3.2)	149 (1.9)		
Ethnicity, *n* (%)	15,431						< 0.001	< 0.001
Non‐hispanic		13,237 (86)	2811 (87)	676 (86)	2748 (79)	7002 (88)		
Hispanic		2194 (14)	418 (13)	107 (14)	742 (21)	927 (12)		
Insurance type, *n* (%)	17,596						< 0.001	< 0.001
Public		7324 (42)	1741 (48)	376 (43)	2076 (52)	3131 (34)		
Private		9922 (56)	1845 (50)	491 (56)	1816 (46)	5770 (64)		
Other		350 (2.0)	68 (1.9)	11 (1.3)	91 (2.3)	180 (2.0)		
Study site, *n* (%)	18,672						< 0.001	< 0.001
North Carolina		4766 (26)	1152 (29)	237 (25)	862 (21)	2515 (26)		
Colorado		4707 (25)	834 (21)	249 (27)	1111 (26)	2513 (26)		
New York		4570 (24)	843 (22)	207 (22)	1251 (30)	2269 (24)		
Utah		2759 (15)	536 (14)	125 (13)	543 (13)	1555 (16)		
Georgia		1870 (10)	546 (14)	112 (12)	430 (10)	782 (8.1)		
Charlson comorbidity index, *n* (%)	18,672						< 0.001	< 0.001
0		9289 (50)	2181 (56)	470 (51)	2198 (52)	4440 (46)		
1		4543 (24)	857 (22)	232 (25)	968 (23)	2486 (26)		
2		2259 (12)	449 (11)	114 (12)	493 (12)	1203 (12)		
3 or more		2581 (14)	424 (11)	114 (12)	538 (13)	1505 (16)		

*Note: N* excludes missing (race, *n* = 3457; ethnicity, *n* = 3241; insurance type, *n* = 1076; sex, *n* = 4). See supplemental file for missing data analysis. Except for age group and Charlson score, values sorted by descending frequency.

^a^ Kruskal‐Wallis rank sum test; Pearson's Chi‐squared test.

^b^ False discovery rate correction for multiple testing.

Median age was 38 years overall and varied between CHD groups (*p* < 0.001). The severe CHD group had the lowest median age (30 years) (Table 1). Figure S2 illustrates the skewed age distribution in the CHD groups (all pairwise contrasts were associated with *p* < 0.001). Additionally, 52% of the overall cohort were women, 21% were of a race other than white, 42% had public insurance, and 50% had a Charlson comorbidity index of 1 or greater. All characteristics were differentially distributed across CHD severity groups.

### 3.2 Comorbidity and Multimorbidity

Among the 18,672 adults with CHD in the cohort, 89% (*n* = 16,256) had at least one comorbidity, and 76% (*n* = 14,155) had multiple (2 or more) comorbidities. Table 2 summarizes the cohort characteristics associated with the presence and number of comorbidities.

**TABLE 2:** Factors associated with the presence and number of comorbidities in adults 19–64 years old, CHD lifespan study, 2011–2013.

Characteristic	With comorbidity			Adj IRR for having a comorbidity				Adj IRR for number of comorbidities		
	*N*	Yes	No	*N*	IRR (95% CI)^a^	*p*	*q*‐value^b^	IRR (95% CI)^a^	*p*	*q*‐value^b^
Age group, *n* (%)	18,672	16,526 (88.5)	2146 (11.5)	12,964		< 0.001	< 0.001		< 0.001	< 0.001
19–24		2819 (78)	809 (22)	2267	1.00			1.00		
25–34		3740 (84)	709 (16)	3014	1.06 (1.04–1.09)	< 0.001	< 0.001	1.33 (1.28–1.39)	< 0.001	< 0.001
35–44		2987 (90)	316 (9.6)	2334	1.13 (1.10–1.15)	< 0.001	< 0.001	1.77 (1.69–1.85)	< 0.001	< 0.001
45–54		3434 (95)	195 (5.4)	2670	1.17 (1.15–1.20)	< 0.001	< 0.001	2.29 (2.20–2.39)	< 0.001	< 0.001
55–64		3546 (97)	117 (3.2)	2679	1.18 (1.16–1.21)	< 0.001	< 0.001	2.71 (2.60–2.83)	< 0.001	< 0.001
Sex, *n* (%)	18,668			12,964		0.53	0.58		< 0.001	< 0.001
Female		8413 (87)	1252 (13)	6686	1.00			1.00		
Male		8109 (90)	894 (9.9)	6278	1.01 (1.00–1.02)	0.019	0.032	1.07 (1.04–1.10)	< 0.001	< 0.001
Unknown		4	0							
Race, *n* (%)	15,215			12,964		0.026	0.038		< 0.001	< 0.001
White		10,861 (90)	1158 (9.6)	10,189	1.00			1.00		
Black		2147 (92)	184 (7.9)	1972	1.03 (1.02–1.04)	< 0.001	< 0.001	1.08 (1.04–1.12)	< 0.001	< 0.001
Other		403 (80)	103 (20)	456	0.87 (0.83–0.91)	< 0.001	< 0.001	0.69 (0.64–0.74)	< 0.001	< 0.001
Multiracial		345 (96)	14 (3.9)	347	1.05 (1.03–1.07)	< 0.001	< 0.001	1.24 (1.16–1.34)	< 0.001	< 0.001
Unknown		2770	687							
Ethnicity, *n* (%)	15,431			12,964		0.57	0.59		< 0.001	< 0.001
Non‐hispanic		12,049 (91)	1188 (9.0)	11,928	1.00			1.00		
Hispanic		2017 (92)	177 (8.1)	1036	1.02 (1.00–1.04)	0.023	0.035	1.08 (1.03–1.13)	< 0.001	< 0.001
Unknown		2460	781							
Insurance type, *n* (%)	17,596			12,964		< 0.001	< 0.001		< 0.001	< 0.001
Public		6803 (93)	521 (7.1)	5459	1.00			1.00		
Private		8548 (86)	1374 (14)	7269	0.92 (0.91–0.93)	< 0.001	< 0.001	0.57 (0.55–0.58)	< 0.001	< 0.001
Other		294 (84)	56 (16)	236	0.92 (0.88–0.96)	< 0.001	< 0.001	0.55 (0.50–0.60)	< 0.001	< 0.001
Unknown		881	195							
Site, *n* (%)	18,672			12,964		0.028	0.038		< 0.001	< 0.001
North Carolina		4210 (88)	556 (12)	3125	1.00			1.00		
Colorado		3903 (83)	804 (17)	3045	0.95 (0.93–0.96)	< 0.001	< 0.001	0.86 (0.83–0.90)	< 0.001	< 0.001
New York		4198 (92)	372 (8.1)	3645	0.94 (0.93–0.95)	< 0.001	< 0.001	0.87 (0.84–0.90)	< 0.001	< 0.001
Utah		2607 (94)	152 (5.5)	1845	1.01 (1.00–1.03)	0.049	0.062	1.08 (1.04–1.13)	< 0.001	< 0.001
Georgia		1608 (86)	262 (14)	1304	0.94 (0.92–0.96)	< 0.001	< 0.001	0.94 (0.90–0.98)	0.009	0.010
Severity class, *n* (%)	18,672			12,964		0.78	0.78		< 0.001	< 0.001
Severe		3398 (87)	513 (13)	2730	1.00			1.00		
Shunt‐valve		820 (88)	110 (12)	661	1.01 (0.99–1.04)	0.28	0.33	1.07 (1.00–1.13)	0.037	0.038
Shunt		3571 (85)	626 (15)	2783	0.98 (0.96–1.00)	0.032	0.043	0.91 (0.88–0.95)	< 0.001	< 0.001
Valve		8737 (91)	897 (9.3)	6790	1.00 (0.99–1.02)	0.53	0.58	0.98 (0.95–1.02)	0.28	0.28

*Note: N* reflects the effective number of cases in the multivariable analyses (i.e., excludes missing values across all covariates); see supplemental file for missing data analysis. Adjusted IRR for having a comorbidity was estimated via Poisson regression models with robust error variance, adjusted for age group, sex, race, ethnicity, study site, and insurance type (see methods for details). Adjusted IRR for the number of comorbidities was estimated via negative binomial regression models, adjusted for the same covariates.

^a^ CI, confidence interval; IRR, incidence rate ratio.

^b^ False discovery rate correction for multiple testing.

**FIGURE 1:**
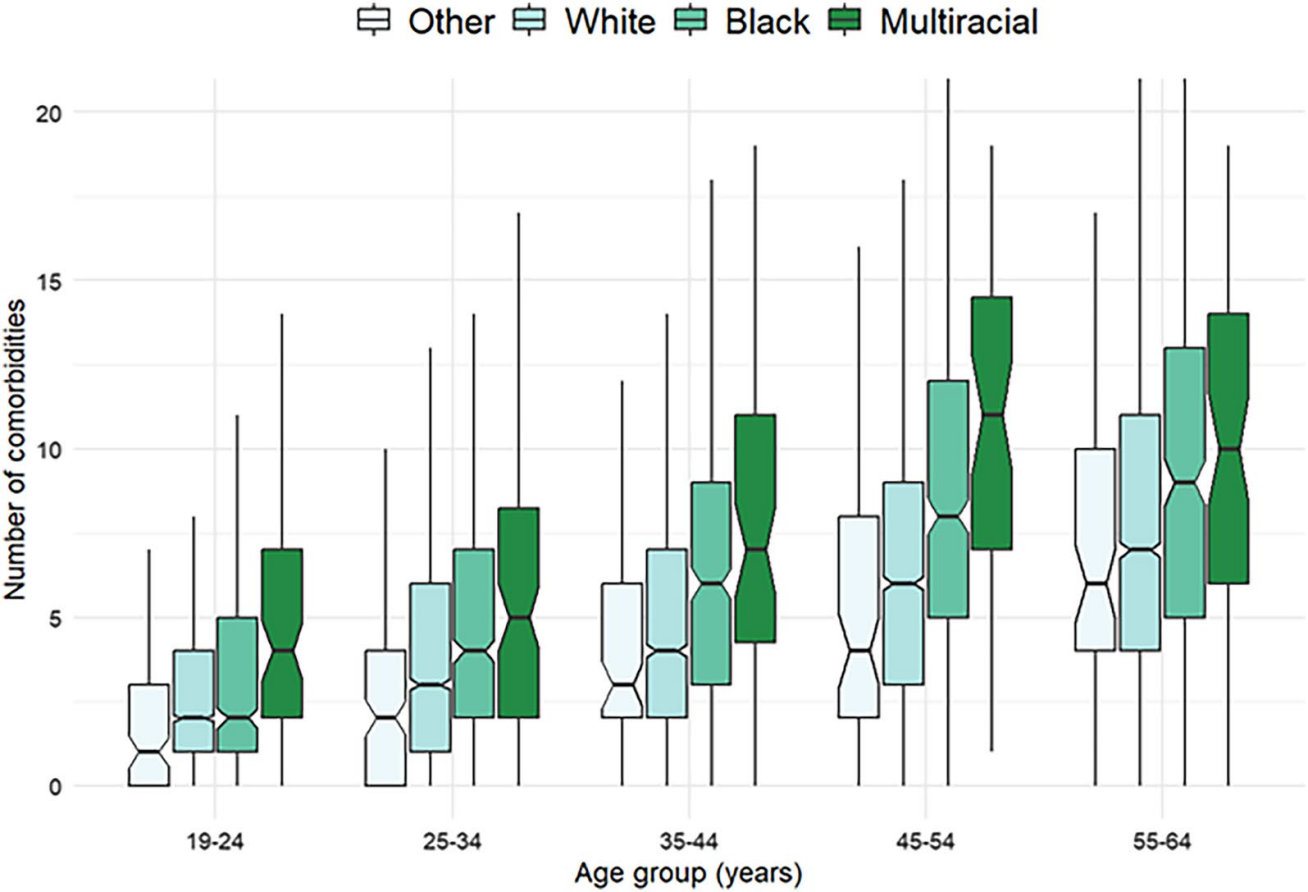
Number of comorbidities by race and by age, Lifespan study, 2011–2013. Note: The notch in the boxplot spans the 95% confidence interval of the median (bold horizontal line).

Several factors, including age, were associated with the presence of comorbidity and with an increasing number of comorbidities (Table 2). The incidence of multimorbidity (having multiple comorbidities) increased 2.7‐fold from the youngest age group (19–24 years) to the oldest age group (55 to 64 years old). Other sociodemographic factors associated with multimorbidity included male sex (compared to female), Black and multiracial race (compared to White), and Hispanic ethnicity (compared to non‐Hispanic). The greater multimorbidity among Black and multiracial adults was observed in all age groups (Figure 1). Such a pattern of higher multimorbidity in select race groups was observed across CHD groups (Figure S3).

Having private insurance was associated with a lower prevalence of multimorbidity compared to public insurance. The relation of multimorbidity with CHD groups was less clear, with only shunt lesions having a lower rate of multimorbidity than severe CHD (Table 2).

### 3.3 Types and Patterns of Comorbidities

Table 3 (with additional details in Table S1) summarizes the frequency of comorbidity groups (e.g., neurologic, endocrine) and comorbidity patterns (e.g., cardiac and noncardiac) by CHD group and age. Comorbidities (range, 78%–96%), including both cardiac (39%–85%) and noncardiac (71%–89%), were highly prevalent overall, in both sexes, and across CHD groups within each age group.

**TABLE 3:** Proportion (%) of adults 19–64 years old with congenital heart disease (CHD) with selected major comorbidities and comorbidity patterns, by age group and sex, lifespan study, 2011–2013.

Comorbidity	Overall and by sex			< 40 years				40 years and above			
	Overall, *N* = 18,672	Female, *N* = 9665	Male, *N* = 9003	Severe, *N* = 2900	Shunt‐valve	Shunt	Valve, *N* = 3898	Severe, *N* = 1011	Shunt‐valve, *N* = 354	Shunt, *N* = 1788	Valve, *N* = 5736
					*N* = 576	*N* = 2409					
Any comorbidity	89	87	90	85	84	78	83	92	96	94	96
Multiple comorbidities	76	74	78	68	69	63	64	82	91	86	88
Cardiac comorbidity	67	61	73	65	65	39	61	78	85	65	81
Non‐cardiac comorbidity	79	79	79	71	72	72	69	82	91	91	89
Cardiac comorbidities											
Residual cardiac disease	48	43	54	44	51	19	49	51	69	30	66
Arrhythmia	30	27	33	38	27	18	18	51	46	33	34
Heart failure	19	17	22	24	15	9.4	10	36	31	22	24
Coronary artery disease	16	12	20	7.4	7.1	6.4	5.4	20	28	24	28
Aortic disease	13	8	17	5	6.9	1.4	15	7	18	4.2	24
Other cardiovascular disease	10	9.2	11	8.5	10	6.3	7.3	13	19	11	13
Other vascular disease	10	9.9	10	6.7	6.1	6.4	5	14	19	16	14
Non‐cardiac comorbidities											
Psychiatric	38	37	39	29	32	34	31	41	48	48	47
Endocrine	37	36	39	17	19	21	18	46	54	59	60
Hypertension	35	30	41	14	17	14	21	42	53	53	59
Hematologic	29	29	29	23	21	24	18	39	48	36	38
Other pulmonary	28	27	29	24	23	19	16	39	50	37	36
Gastrointestinal	27	27	26	17	16	22	17	34	37	37	36
Obstructive pulmonary	22	23	22	15	15	18	15	26	32	32	30
Nutrition	19	22	16	14	16	19	12	21	27	26	24
Neurologic	18	18	19	14	15	15	11	25	31	30	23
Oncologic	16	18	15	8.4	8	10	8.9	20	22	26	25
Renal	13	11	15	7.5	5.4	6.6	7	18	16	20	19
Orthopedic	12	13	11	5.3	5.7	5.3	5.2	16	17	21	19
Hepatic	11	10	12	11	6.6	6.1	6.5	16	15	16	14
Pulmonary vascular	5.7	6.6	4.8	6	4.9	5.2	3	11	13	10	5.1
Genetic syndromes	4.3	4.3	4.4	9.8	6.9	7.5	2.4	5.5	3.1	4.5	1.1
Immunologic	3.2	4.3	2	2.6	1.4	2.3	2.1	3.8	5.4	4.5	4.3
Learning or intellectual	2.6	2.1	3.1	3.4	4.9	3.6	2.7	2.6	2.3	2.5	1.5

*Note:* Shading intensity correlates with the proportion of individuals in each cell. Proportions are column proportions.

Noncardiac comorbidities were more frequent than cardiac comorbidities. Generally, comorbidities were more prevalent among those 40 years and above than those younger than 40. Men had higher rates of cardiac comorbidity (including heart failure, coronary heart disease, and aortic disease) and hypertension compared to women. In contrast, women had a higher rate of pulmonary vascular disease.

Aside from residual cardiac disease, present in approximately half of the cohort overall, other highly prevalent cardiac comorbidities (Table 3) were heart failure (9.4%–24% among those < 40 years of age depending on CHD type; 22%–36% among those 40 years of age and older); arrhythmias (18%–38% in the younger age group; 33%–51% in the older age group); and coronary artery disease, particularly among the older age group (20%–28%, depending on CHD type).

Highly prevalent (i.e., > 30%) noncardiac comorbidities included psychiatric conditions, primarily depression and anxiety disorders, reported in nearly one‐third of the cohort younger than 40 years of age and half of the cohort aged 40 years or older, fairly uniformly across all CHD types; endocrine disorders, including diabetes, dyslipidemia, and thyroid disease, reported in about 20% of the cohort 19–40 years of age and half or more across CHD types in the cohort aged 40 years and older; and systemic hypertension, in approximately half of the cohort in the older age group, particularly among the nonsevere CHD groups (shunt, valve, shunt‐valve).

Genetic syndromes (Table 3) were reported in 4.3% of this adult cohort (*n* = 807). Of these syndrome diagnoses, 90% (*n* = 724) were chromosomal anomalies, including Down syndrome (78.2% of all syndrome diagnoses), deletion 22q11 syndrome (8.3%), and other chromosomal trisomies and deletions (3.2%). Among individuals with a syndrome diagnosis, 42.3% (*n* = 341) had a severe CHD, including 44.2% of those with Down syndrome (*n* = 279) and 74.6% (*n* = 50) with deletion 22q11 syndrome.

Comorbidity also varied by other demographic variables (Table 4). Individuals reported as Black and multiracial, compared to White, had a small but significant excess prevalence of comorbidity, multimorbidity, and non‐cardiac comorbidity. A similar excess was seen in most cardiac comorbidity subgroups, except for aortic disease (−9.2%) and residual cardiac disease (−6.4%). Comorbidities with the largest excess associated with multiracial and Black race included hypertension (+12%), heart failure (+11%), renal disease (+9.3%), obstructive pulmonary disease (+ 5.6%), and coronary artery disease (+5.1%).

**TABLE 4:** Patterns of comorbidity and comorbidity groups by race group in adults with congenital heart disease (CHD), lifespan study, 2011–2013.

Comorbidity type	Black/multiracial *N* = 2690	White *N* = 12,019	Difference (95% CI)^a^, ^b^	*p* ^*a*^
Any comorbidity, *n* (%)	2492 (93)	10,861 (90)	2.3% (1.1%–3.4%)	< 0.001
Multiple comorbidities, *n* (%)	2221 (83)	9375 (78)	4.6% (2.9%–6.2%)	< 0.001
Cardiac comorbidity, *n* (%)	1820 (68)	8483 (71)	−2.9% (−4.9% to −0.95%)	0.002
Non‐cardiac comorbidity, *n* (%)	2347 (87)	9619 (80)	7.2% (5.7%–8.7%)	< 0.001
Cardiac comorbidities				
Heart failure, *n* (%)	802 (30)	2246 (19)	11% (9.2%–13%)	< 0.001
Arrhythmia, *n* (%)	929 (35)	3778 (31)	3.1% (1.1%–5.1%)	0.002
Residual cardiac disease *n* (%)	1236 (46)	6297 (52)	−6.4% (−8.6% to −4.3%)	< 0.001
Coronary artery disease *n* (%)	570 (21)	1932 (16)	5.1% (3.4%–6.8%)	< 0.001
Other cardiovascular disease, *n* (%)	383 (14)	1216 (10)	4.1% (2.7%–5.6%)	< 0.001
Aortic, *n* (%)	160 (5.9)	1815 (15)	−9.2% (−10% to −8.0%)	< 0.001
Other vascular, *n* (%)	398 (15)	1204 (10)	4.8% (3.3%–6.2%)	< 0.001
Noncardiac comorbidities				
Hypertension, *n* (%)	1255 (47)	4223 (35)	12% (9.4%–14%)	< 0.001
Obstructive pulmonary, *n* (%)	766 (28)	2755 (23)	5.6% (3.7%–7.4%)	< 0.001
Pulmonary vascular, *n* (%)	254 (9.4)	646 (5.4)	4.1% (2.9%–5.3%)	< 0.001
Other pulmonary, *n* (%)	949 (35)	3399 (28)	7.0% (5.0%–9.0%)	< 0.001
Renal, *n* (%)	570 (21)	1429 (12)	9.3% (7.6%–11%)	< 0.001
Hepatic, *n* (%)	422 (16)	1315 (11)	4.7% (3.2%–6.3%)	< 0.001
Gastrointestinal, *n* (%)	849 (32)	3312 (28)	4.0% (2.1%–6.0%)	< 0.001
Neurologic, *n* (%)	634 (24)	2263 (19)	4.7% (3.0%–6.5%)	< 0.001
Hematologic, *n* (%)	1081 (40)	3434 (29)	12% (9.6%–14%)	< 0.001
Nutrition, *n* (%)	795 (30)	2111 (18)	12% (10%–14%)	< 0.001
Orthopedic, *n* (%)	364 (14)	1535 (13)	0.76% (−0.69% to 2.2%)	0.30
Endocrine, *n* (%)	1121 (42)	4614 (38)	3.3% (1.2%–5.4%)	0.002
Infection, *n* (%)	1381 (51)	4119 (34)	17% (15%–19%)	< 0.001
Immunologic, *n* (%)	133 (4.9)	384 (3.2)	1.7% (0.85%–2.6%)	< 0.001
Learning or intellectual, *n* (%)	110 (4.1)	305 (2.5)	1.6% (0.73%–2.4%)	< 0.001
Psychiatric, *n* (%)	1234 (46)	4780 (40)	6.1% (4.0%–8.2%)	< 0.001
Genetic syndromes, *n* (%)	128 (4.8)	532 (4.4)	0.33% (−0.58% to 1.2%)	0.48
Oncologic, *n* (%)	494 (18)	2133 (18)	0.62% (−1.0% to 2.3%)	0.47

^a^ Two sample test for equality of proportions.

^b^ CI, confidence interval.

Table 5 focuses on more specific comorbidities of particular clinical relevance. Stroke/transient ischemic attack (TIA) shows a several‐fold difference in prevalence by age group, increasing from 2.5% in the youngest age group (19 to 24 years) to 18% in the oldest age group (55–64 years) (*p* < 0.001). Highly prevalent conditions that markedly increased with age include obstructive pulmonary disease and the cardiometabolic “cluster” of atherosclerotic risk factors (hypertension, type 2 diabetes, and dyslipidemia), as well as oncologic conditions. Depression and anxiety were among the top 5 most prevalent comorbidities within most age groups.

**TABLE 5:** Proportion (%) of individuals with selected specific comorbidities by age, among adults 19–64 years old with congenital heart disease (CHD), Lifespan study, 2011–2013.

Comorbidity	Age group (years)					
	19–24	25–34	35–44	45–54	55–64	*p* ^a^
	*N* = 3628	*N* = 4449	*N* = 3303	*N* = 3629	*N* = 3663	
Hypertension, essential	11	17	33	51	66	< 0.001
Dyslipidemia	2.5	7.7	22	42	59	< 0.001
Coronary artery disease	4.3	6.9	12	22	36	< 0.001
Pulmonary obstructive disease	13	16	20	28	35	< 0.001
Diabetes type 2	1.9	4.4	8.2	16	24	< 0.001
Orthopedic disorder	1.2	2.4	6.3	13	21	< 0.001
Obesity	7.1	11	15	17	19	< 0.001
Depression/bipolar disorder	9.9	13	17	20	19	< 0.001
Stroke/transient ischemic attack	2.5	5	8.6	13	18	< 0.001
Thyroid disease	5	8.1	11	15	17	< 0.001
Anxiety disorder	11	14	18	18	17	< 0.001
Oncologic disease	3.3	5.1	8.2	12	17	< 0.001
Renal disease	1.3	2.6	5	7.4	13	< 0.001
Hepatic disease	2.3	3.8	4.2	5.8	6.8	< 0.001
Alcohol abuse disorder	2.3	2.7	3.5	4.9	4.9	< 0.001
Psychosis—schizophrenia	1.4	2	2.4	3.2	3.1	< 0.001

*Note:* shading intensity correlates with the proportion of individuals in each cell. Proportions are column proportions.

^a^ Pearson's Chi‐squared test.

### 3.4 Missing Data Analysis

Three variables (Table 1) had > 1% missing data: race (18.5%), ethnicity (17.4%), and insurance type (5.8%). Missing data analysis is detailed in the supplemental file. Briefly, there was little to no correlation among these variables. The effect estimates from the original data (Table 2) were similar to those derived from the imputed datasets (see Supporting Information). For this reason, Table 2 shows the original (unimputed) findings.

## 4 Discussion

With better survival, the disease burden over the lifespan in people living with CHD is increasingly shifting away from residual cardiac disease toward acquired cardiovascular and systemic complications (Liu et al. 2023; Lui et al. 2017; Forman et al. 2018). This population‐based study from five areas in the US further delineates these patterns and adds to the growing body of information (see Table S2 for a summary of prior studies).

In this study of more than 18,000 adults with CHD (Table 1), comorbidity (89%) and multimorbidity (76%) were the norm, with non‐cardiac comorbidities exceeding cardiac comorbidities (Table 3). Presence and number of comorbidities were unequally distributed across socio‐demographic factors (Table 2). Compared to women, men had higher overall rates of comorbidity and multimorbidity than women, including for coronary heart disease, heart failure, and hypertension (Table 3). Conversely, women had comparatively higher rates of pulmonary vascular disease (6.6% vs. 4.8%) (Dimopoulos et al. 2014; Van Der Feen et al. 2017; Van Riel et al. 2014). Individuals of Black and multiracial backgrounds experienced higher rates of many comorbidities, both cardiac and non‐cardiac, compared to individuals of White racial background (Table 4). Multimorbidity, as measured by the number of comorbidities, showed a similar pattern persisting across age groups (Figure 1) and CHD types (Figure S3). An exception was aortic disease, which was comparatively lower (−9.2%) in individuals with CHD of Black or multiracial backgrounds (Table 4) (Patel et al. 2014; Czarny et al. 2021).

More generally, age was a major demographic factor markedly associated with comorbidity and multimorbidity (Table 2). Prior studies reported similar findings from various geographic areas and health care systems (Table S2) (Lui et al. 2017; Neidenbach et al. 2018; Maurer et al. 2021; Agarwal et al. 2019). In a registry‐based study of adults with CHD in Germany, for example, the proportion of individuals with comorbidities was nearly 50% higher in those 40 years of age or older compared to those < 40 years (78% vs. 57%), with an incidence rate ratio that increased by an estimated 3% by year of age (Maurer et al. 2021). Although such findings across studies are not directly comparable due to differences in methods and comorbidity classification, the age effect is such that it must be accounted for in analyses of comorbidity. For example, individuals with severe CHD in this cohort were younger on average than individuals with milder CHD (Table 1, Figure S2). Information that is age‐adjusted (Table 2) or age‐stratified (Tables 3 and 5) generates a more accurate profile of the comorbidity experience and risk in people with CHD over the lifespan, which is critically important for policy making and guideline development.

This study also highlights the high prevalence of comorbidity clusters that are both common and largely preventable, including hypertension, diabetes, obesity, and dyslipidemia (Table 5), among people with CHD. These atherosclerotic risk factors for acquired cardiovascular and cerebrovascular complications (e.g., coronary heart disease, myocardial infarction, and stroke) influence overall morbidity and mortality in people with CHD. In this study (Table 5), stroke/TIA was reported in 2.5% of younger individuals with CHD (ages 19–24 years) but nearly four times more frequently (18%) in those aged 55–64 years. Hypertension showed over a six‐fold difference in prevalence between these same age groups (11% vs. 66%). More generally, endocrine/metabolic disorders (including diabetes and hyperlipidemia) and hypertension are consistently within the top 3 non‐cardiac comorbidities in many studies of people living with CHD (Table S2), whether based on data from registries (Maurer et al. 2021), single tertiary care centers (Neidenbach et al. 2018), commercial insurance claims data (Agarwal et al. 2019), or inpatient databases (Singh et al. 2018). Renal disease was another relatively common and clinically significant noncardiac comorbidity (Table 5), as chronic kidney disease has been associated with a threefold increased mortality risk in adults with CHD (Konstantinos Dimopoulos et al. 2008).

Overall, the type of CHD (broadly divided into severe, shunt‐valve, shunt, and valve) influenced, to some degree, the comorbidity frequency, number, and pattern (Table 2). Individuals with severe CHD, compared to those with less severe CHD (shunt and/or valve lesions), experienced higher rates of cardiac comorbidities such as heart failure and arrhythmias, which are typically related to residual cardiac disease (Table 3). However, noncardiac comorbidities were reported relatively uniformly across CHD types, with slightly higher prevalence among some less severe CHD (Table 3). Psychiatric comorbidities (Table 3), including depression and anxiety (Table 5), were common overall and within each age group, highlighting the importance of integrating mental health providers into lifelong congenital cardiac care (Kovacs et al. 2022).

Generalizations based on these findings must consider the study's limitations and strengths. Individuals were identified based on CHD‐coded encounters between 2011 and 2013 within data systems available to investigators and related to a specific period. This approach would have missed comorbidities in individuals with CHD who did not have eligible encounters in the period, had encounters at points of care not captured in the sites' data sources, or had encounters that did not include a CHD code. In general, such events would tend to undercount comorbidities. Also, comorbidities were characterized based on ICD‐9 codes, rather than clinical notes. Findings from a validity study from this same dataset, comparing administrative codes with medical record review in a sample of cases, suggest reasonable validity of diagnostic CHD codes.^17^ Nevertheless, code misclassification cannot be excluded. Non‐differential misclassification, on average, would have led to a decrease in the differences between groups, suggesting that the reported differences (e.g., by severity or demographic characteristics) are minimal estimates. The effect of differential misclassification (e.g., by race, ethnicity, or CHD group) would be more difficult to predict.

Strengths of the study include the population‐based design, the inclusion of geographically and socio‐demographically diverse study areas, and the availability of outpatient/ambulatory data in addition to inpatient stays. These findings add to previous studies in the US that evaluated more restricted groups of adults with CHD, such as inpatients only (National Inpatient Database) (Singh et al. 2018) or members of a specific commercial health insurance system (IBM Marketscan Claims Data) (Agarwal et al. 2019). The cross‐sectional study design was both a limitation and a strength of the study. Whereas the design does not allow for reconstructing the longitudinal health trajectory of a specific cohort, the findings in a cross‐sectional study closely reflect the population's present comorbidity experience originating from health histories and patterns of care from earlier periods, when the focus on early prevention might have been less rigorous. Such information is crucial as this population, comprising interwoven cohorts of people of different ages and health histories, is the primary target and beneficiary of policies, treatments, and care guidelines.

From a broader perspective, the epidemiologic shift driven by more prolonged survival in an aging population has prompted a call to specifically address the prevention, identification, and care of comorbidity and multimorbidity when developing management guidelines to improve outcomes in adults with CHD (Liu et al. 2023; Lui et al. 2017; Forman et al. 2018). Current (2018) US guidelines (Stout et al. 2019) devote limited attention to noncardiac comorbidities, mainly encouraging clinicians to address these in their care programs. An emerging approach, arguably more integrated and reflective of clinical reality, aims to address the frequency of comorbidities and their co‐occurrence in multimorbidity clusters (Forman et al. 2018; Skou et al. 2022). Multimorbidity, which our data and those from others indicate as the norm rather than the exception, presents challenges (Skou et al. 2022; Ho et al. 2021) but also opportunities for better prevention and care, including for mental health (Kovacs et al. 2022), if explicitly addressed by practice guidelines, clinical programs, and research priorities (Forman et al. 2018).

In summary, this multi‐site, population‐based study highlights the substantial burden of comorbidity and multimorbidity in adults with congenital heart disease, including preventable and manageable conditions such as diabetes, obesity, and hypertension. If left undiagnosed or inadequately treated, these conditions can exacerbate cardiovascular and cerebrovascular complications in an already vulnerable population. The findings also emphasize variability in morbidity and multimorbidity across demographic subgroups, including. The strong association with age suggests that, without targeted prevention and intervention, the impact of comorbidity will likely intensify as the population ages. These findings may inform strategies to enhance treatment and preventive care for adults with CHD throughout their lifespan. Given the consistent patterns of comorbidity across studies, geographic regions, and healthcare systems, developing integrated guidelines that address both comorbidity and multimorbidity in adults with CHD, grounded in real‐world population health metrics, may prove broadly applicable and impactful.

## Author Contributions

Dr. Lorenzo D. Botto and Dr. Matthew R. Reeder had full access to all data in the study and take responsibility for the integrity of the data and the accuracy of the results. **Lorenzo D. Botto, Jill Glidewell, Wendy M. Book, Tessa L. Crume, Aida S. Soim, George K. Lui, Jennifer S. Li:** concept and design. **Lorenzo D. Botto, Matthew R. Reeder, Jill Glidewell, Wendy M. Book, Tessa L. Crume, Sergey Krikov, Jesse M. DeLaRosa, Karen Chiswell, Jennifer S. Li:** acquisition, analysis, or interpretation of the data. **Lorenzo D. Botto, Feldkamp, Pinto, Whitehead, Jennifer S. Li:** drafting of the manuscript. **All authors:** critical review of the manuscript for important intellectual content. **Lorenzo D. Botto, Matthew R. Reeder, Feldkamp, Jesse M. DeLaRosa, Karen Chiswell:** statistical analysis. **Matthew R. Reeder, Jill Glidewell, Feldkamp, Downing, Jesse M. DeLaRosa, Karen Chiswell:** administrative, Technical, or Material Support. **Lorenzo D. Botto, Jill Glidewell, Wendy M. Book, Tessa L. Crume, Aida S. Soim, Li:** supervision.

## Disclosure

The findings and conclusions in this report are those of the authors and do not necessarily represent the official position of the Centers for Disease Control and Prevention. This analysis has undergone replication by Jesse Delarosa, MStat, and Karen Chiswell, PhD.

## Ethics Statement

The Institutional Review Boards from Emory University in Georgia (GA), the New York State Department of Health (NY), Duke University in North Carolina (NC), the University of Colorado (CO), and the University of Utah (UT) approved an analysis of de‐identified data. Each site's Institutional Review Board waived the requirement for informed consent due to the de‐identified data analysis.

## Conflicts of Interest

The authors declare no conflicts of interest.

## Supporting information


**Data S1.** Supporting Information.

**Data S2.** Supporting Information.

## Data Availability

The data that support the findings of this study are available on request from the corresponding author. The data are not publicly available due to privacy or ethical restrictions.

## References

[bdr22515-bib-0001] Agarwal, A. , R. Thombley , C. S. Broberg , et al. 2019. “Age‐ and Lesion‐Related Comorbidity Burden Among US Adults With Congenital Heart Disease: A Population‐Based Study.” Journal of the American Heart Association 8, no. 20: e013450. 10.1161/JAHA.119.013450. 31575318 PMC6818026

[bdr22515-bib-0002] AHRQ . 2023. “Clinical Classifications Software (CCS) for ICD‐9‐CM.” https://hcup‐us.ahrq.gov/toolssoftware/ccs/ccs.jsp.

[bdr22515-bib-0003] Billett, J. , M. R. Cowie , M. A. Gatzoulis , I. F. Vonder Muhll , and A. Majeed . 2008. “Comorbidity, Healthcare Utilisation and Process of Care Measures in Patients With Congenital Heart Disease in the UK: Cross‐Sectional, Population‐Based Study With Case‐Control Analysis.” Heart 94, no. 9: 1194–1199. 10.1136/hrt.2007.122671. 17646191

[bdr22515-bib-0004] Bracher, I. , M. Padrutt , F. Bonassin , et al. 2017. “Burden and Impact of Congenital Syndromes and Comorbidities Among Adults With Congenital Heart Disease.” International Journal of Cardiology 240: 159–164. 10.1016/j.ijcard.2017.02.118. 28606676

[bdr22515-bib-0005] Charlson, M. E. , D. Carrozzino , J. Guidi , and C. Patierno . 2022. “Charlson Comorbidity Index: A Critical Review of Clinimetric Properties.” Psychotherapy and Psychosomatics 91, no. 1: 8–35. 10.1159/000521288. 34991091

[bdr22515-bib-0006] Charlson, M. E. , P. Pompei , K. L. Ales , and C. R. MacKenzie . 1987. “A New Method of Classifying Prognostic Comorbidity in Longitudinal Studies: Development and Validation.” Journal of Chronic Diseases 40, no. 5: 373–383. 10.1016/0021-9681(87)90171-8. 3558716

[bdr22515-bib-0007] Czarny, M. J. , S. J. Shah , S. P. Whelton , et al. 2021. “Race/Ethnicity and Prevalence of Aortic Stenosis by Echocardiography in the Multi‐Ethnic Study of Atherosclerosis.” Journal of the American College of Cardiology 78, no. 2: 195–197. 10.1016/j.jacc.2021.04.078. 33989712 PMC8282359

[bdr22515-bib-0008] Diller, G.‐P. , A. Arvanitaki , A. R. Opotowsky , et al. 2021. “Lifespan Perspective on Congenital Heart Disease Research.” Journal of the American College of Cardiology 77, no. 17: 2219–2235. 10.1016/j.jacc.2021.03.012. 33926659

[bdr22515-bib-0010] Dimopoulos, K. , G.‐P. Diller , E. Koltsida , et al. 2008. “Prevalence, Predictors, and Prognostic Value of Renal Dysfunction in Adults With Congenital Heart Disease.” Circulation 117, no. 18: 2320–2328. 10.1161/CIRCULATIONAHA.107.734921. 18443238

[bdr22515-bib-0009] Dimopoulos, K. , S. J. Wort , and M. A. Gatzoulis . 2014. “Pulmonary Hypertension Related to Congenital Heart Disease: A Call for Action.” European Heart Journal 35, no. 11: 691–700. 10.1093/eurheartj/eht437. 24168793

[bdr22515-bib-0011] Forman, D. E. , M. S. Maurer , C. Boyd , et al. 2018. “Multimorbidity in Older Adults With Cardiovascular Disease.” Journal of the American College of Cardiology 71, no. 19: 2149–2161. 10.1016/j.jacc.2018.03.022. 29747836 PMC6028235

[bdr22515-bib-0012] Garmany, A. , S. Yamada , and A. Terzic . 2021. “Longevity Leap: Mind the Healthspan Gap.” npj Regenerative Medicine 6, no. 1: 57. 10.1038/s41536-021-00169-5. 34556664 PMC8460831

[bdr22515-bib-0013] Gilboa, S. M. , O. J. Devine , J. E. Kucik , et al. 2016. “Congenital Heart Defects in the United States: Estimating the Magnitude of the Affected Population in 2010.” Circulation 134, no. 2: 101–109. 10.1161/CIRCULATIONAHA.115.019307. 27382105 PMC4942347

[bdr22515-bib-0014] Glidewell, J. , W. Book , C. Raskind‐Hood , et al. 2018. “Population‐Based Surveillance of Congenital Heart Defects Among Adolescents and Adults: Surveillance Methodology.” Birth Defects Research 110, no. 19: 1395–1403. 10.1002/bdr2.1400. 30394691 PMC6701175

[bdr22515-bib-0016] Ho, I. S.‐S. , A. Azcoaga‐Lorenzo , A. Akbari , et al. 2021. “Examining Variation in the Measurement of Multimorbidity in Research: A Systematic Review of 566 Studies.” Lancet Public Health 6, no. 8: e587–e597. 10.1016/S2468-2667(21)00107-9. 34166630

[bdr22515-bib-0017] Jill Glidewell, M. , S. L. Farr , W. M. Book , et al. 2021. “Individuals Aged 1‐64 Years With Documented Congenital Heart Defects at Healthcare Encounters, Five U.S. Surveillance Sites, 2011‐2013.” American Heart Journal 238, no. August: 100–108. 10.1016/j.ahj.2021.04.007. 33951414 PMC9087052

[bdr22515-bib-0018] Kovacs, A. H. , J. Brouillette , P. Ibeziako , et al. 2022. “Psychological Outcomes and Interventions for Individuals With Congenital Heart Disease: A Scientific Statement From the American Heart Association.” Circulation. Cardiovascular Quality and Outcomes 15, no. 8: e000110. 10.1161/HCQ.0000000000000110. 35862009

[bdr22515-bib-0019] Liu, A. , G.‐P. Diller , P. Moons , C. J. Daniels , K. J. Jenkins , and A. Marelli . 2023. “Changing Epidemiology of Congenital Heart Disease: Effect on Outcomes and Quality of Care in Adults.” Nature Reviews Cardiology 20, no. 2: 126–137. 10.1038/s41569-022-00749-y. 36045220

[bdr22515-bib-0020] Lui, G. K. , A. Saidi , A. B. Bhatt , et al. 2017. “Diagnosis and Management of Noncardiac Complications in Adults With Congenital Heart Disease: A Scientific Statement From the American Heart Association.” Circulation 136, no. 20: e348–e392. 10.1161/CIR.0000000000000535. 28993401

[bdr22515-bib-0021] Marelli, A. J. , R. Ionescu‐Ittu , A. S. Mackie , L. Guo , N. Dendukuri , and M. Kaouache . 2014. “Lifetime Prevalence of Congenital Heart Disease in the General Population From 2000 to 2010.” Circulation 130, no. 9: 749–756. 10.1161/CIRCULATIONAHA.113.008396. 24944314

[bdr22515-bib-0022] Maurer, S. J. , U. M. M. Bauer , H. Baumgartner , A. Uebing , C. Walther , and O. Tutarel . 2021. “Acquired Comorbidities in Adults With Congenital Heart Disease: An Analysis of the German National Register for Congenital Heart Defects.” Journal of Clinical Medicine 10, no. 2: 314. 10.3390/jcm10020314. 33467024 PMC7830982

[bdr22515-bib-0023] Neidenbach, R. C. , E. Lummert , M. Vigl , et al. 2018. “Non‐Cardiac Comorbidities in Adults With Inherited and Congenital Heart Disease: Report From a Single Center Experience of More Than 800 Consecutive Patients.” Cardiovascular Diagnosis and Therapy 8, no. 4: 423–431. 10.21037/cdt.2018.03.11. 30214857 PMC6129827

[bdr22515-bib-0024] Oster, M. E. , A. P. Riser , J. G. Andrews , et al. 2021. “Comorbidities Among Young Adults With Congenital Heart Defects: Results From the Congenital Heart Survey to Recognize Outcomes, Needs, and Well‐being—Arizona, Arkansas, and Metropolitan Atlanta, 2016–2019.” MMWR. Morbidity and Mortality Weekly Report 70, no. 6: 197–201. 10.15585/mmwr.mm7006a3. 33571179 PMC7877580

[bdr22515-bib-0025] Patel, D. K. , K. D. Green , M. Fudim , F. E. Harrell , T. J. Wang , and M. A. Robbins . 2014. “Racial Differences in the Prevalence of Severe Aortic Stenosis.” Journal of the American Heart Association 3, no. 3: e000879. 10.1161/JAHA.114.000879. 24870936 PMC4309086

[bdr22515-bib-0026] Schafer, J. L. 1999. “Multiple Imputation: A Primer.” Statistical Methods in Medical Research 8, no. 1: 3–15. 10.1177/096228029900800102. 10347857

[bdr22515-bib-0027] Singh, S. , R. Desai , H. K. Fong , A. Sadolikar , S. Samani , and H. Goyal . 2018. “Extra‐Cardiac Comorbidities or Complications in Adults With Congenital Heart Disease: A Nationwide Inpatient Experience in the United States.” Cardiovascular Diagnosis and Therapy 8, no. 6: 814–819. 10.21037/cdt.2018.09.12. 30740330 PMC6331373

[bdr22515-bib-0028] Skou, S. T. , F. S. Mair , M. Fortin , et al. 2022. “Multimorbidity.” Nature Reviews Disease Primers 8, no. 1: 48. 10.1038/s41572-022-00376-4. PMC7613517 35835758

[bdr22515-bib-0029] Stout, K. K. , C. J. Daniels , J. A. Aboulhosn , et al. 2019. “2018 AHA/ACC Guideline for the Management of Adults With Congenital Heart Disease: A Report of the American College of Cardiology/American Heart Association Task Force on Clinical Practice Guidelines.” Circulation 139, no. 14: e698–e800. 10.1161/CIR.0000000000000603. 30586767

[bdr22515-bib-0015] Van Buuren, S. , and K. Groothuis‐Oudshoorn . 2011. “Mice: Multivariate Imputation by Chained Equations in R.” Journal of Statistical Software 45, no. 3: 1–67. 10.18637/jss.v045.i03.

[bdr22515-bib-0030] Van Der Feen, D. E. , B. Bartelds , R. A. De Boer , and R. M. F. Berger . 2017. “Pulmonary Arterial Hypertension in Congenital Heart Disease: Translational Opportunities to Study the Reversibility of Pulmonary Vascular Disease.” European Heart Journal 38, no. 26: 2034–2041. 10.1093/eurheartj/ehx034. 28369399

[bdr22515-bib-0031] Van Riel, A. C. M. J. , M. J. Schuuring , I. D. Van Hessen , et al. 2014. “Contemporary Prevalence of Pulmonary Arterial Hypertension in Adult Congenital Heart Disease Following the Updated Clinical Classification.” International Journal of Cardiology 174, no. 2: 299–305. 10.1016/j.ijcard.2014.04.072. 24794056

[bdr22515-bib-0032] Warnes, C. A. , R. Liberthson , G. K. Danielson , et al. 2001. “Task Force 1: The Changing Profile of Congenital Heart Disease in Adult Life.” Journal of the American College of Cardiology 37, no. 5: 1170–1175. 10.1016/S0735-1097(01)01272-4. 11300418

[bdr22515-bib-0033] Yang, H.‐L. , N.‐T. Chang , J.‐K. Wang , C.‐W. Lu , Y.‐C. Huang , and P. Moons . 2020. “Comorbidity as a Mediator of Depression in Adults With Congenital Heart Disease: A Population‐Based Cohort Study.” European Journal of Cardiovascular Nursing 19, no. 8: 732–739. 10.1177/1474515120923785. 32429700

