# Supplementary material for: Comorbidity and Multimorbidity in Adults With Congenital Heart Disease: Findings From a Multi‐Site Population‐Based Study
Source: Birth Defects Res. 2025 Aug 21;117(8):e2515. doi: 10.1002/bdr2.2515 (PMC12368762; doi:10.1002/bdr2.2515)
Supplement: Supplementary file 1 — Data S1. Supporting Information. [file BDR2-117-e2515-s001.docx]

**Supplemental File**

1. **Methods**
2. **Results (tables and figures)**
3. **Missing Data Analysis**
4. **Methods**

**A1. Geographic areas of residence**

Counties included in Georgia: Clayton, Cobb, DeKalb, Fulton, and Gwinnett counties (all in metropolitan Atlanta).

Counties included in New York State: Allegany, Cattaraugus, Chautauqua, Erie, Genesee, Monroe, Niagara, Orleans, and Wyoming in the west and Bronx and Westchester in the south.

Colorado, North Carolina: entire state

**A2. Assignment algorithm for the congenital heart Disease (CHD) groups**

CGD groups were assigned using a predefined stepwise process. Each case was assigned to a CHD group based on ICD-9-CM codes from the linked datasets. When an individual had multiple CHD codes that would map to more than one CHD group, a hierarchical algorithm (below) was used to assign a single CHD group.

Step 1. Assign severity group based on ICD-9-CM codes.

The initial assignment was based on the table below.

| **Severity Group** | **Severity Code** | **CHD type** | **ICD-9-CM code** | **ICD-9-CM_code description** |  |
| --- | --- | --- | --- | --- | --- |
|  |  |  |  |  |  |
| Severe | 1 | Truncus | 745 | Common Truncus |  |
| Severe | 1 | Transposition Complex | 745.1 | Transposition of the Great Arteries (TGA) |  |
| Severe | 1 | Transposition Complex | 745.1 | Complete TGA (dextro-TGA), NOS or classical |  |
| Severe | 1 | Transposition Complex | 745.11 | DORV, or incomplete TGA |  |
| Severe | 1 | Transposition Complex | 745.12 | Corrected TGA (levo-TGA) |  |
| Severe | 1 | Transposition Complex | 745.19 | TGA OS |  |
| Severe | 1 | Tetralogy of Fallot | 745.2 | Tetralogy of Fallot |  |
| Severe | 1 | Univentricular Heart | 745.3 | Single Ventricle, or cor triloculare |  |
| Severe | 1 | Atrioventricular Canal Defects | 745.6 | Endocardial Cushion Defect (aka AVSD) |  |
| Severe | 1 | Atrioventricular Canal Defects | 745.6 | Endocardial Cushion Defect (aka AVSD) unspec. |  |
| Severe | 1 | Atrioventricular Canal Defects | 745.69 | Endocardial Cushion Defect (aka AVSD) Other |  |
| Severe | 1 | Pulmonary Valve Atresia | 746.01 | Pulmonary valve atresia or absence |  |
| Severe | 1 | Tricuspid Atresia | 746.1 | Tricuspid atresia, stenosis or absence |  |
| Severe | 1 | Hypoplastic Left Heart Syndrome | 746.7 | HLHS (hypoplastic left heart syndrome) |  |
| Severe | 1 | Interrupted Aortic Arch | 747.11 | Interrupted aortic arch |  |
| Severe | 1 | TAPVR | 747.41 | Total anomalous pulmonary venous return (TAPVR) |  |
| Shunts | 2 | Ventricular Septal Defect | 745.4 | VSD |  |
| Shunts | 2 | Atrial Septal Defect | 745.5 | ASD2 or PFO |  |
| Shunts | 2 | Atrioventricular Canal Defects | 745.61 | ASD-1 (primum) |  |
| Shunts | 2 | Other Septal closure | 745.8 | Other specified defect of septal closure |  |
| Shunts | 2 | Other Septal closure | 745.9 | Unspecified defect of septal closure |  |
| Shunts | 2 | PDA | 747 | PDA |  |
| Shunts | 2 | Anomalies of Great Veins | 747.42 | Partial anomalous venous return (PAPVR) |  |
| Shunt+valve | 3 | Case has Shunt AND Valve codes |  | (reflects labels of ICD codes of the combination) |  |
| Valve | 4 | Pulmonary Valve | 746 | Anomalies of pulmonary valve |  |
| Valve | 4 | Pulmonary Valve | 746 | Pulmonary valve anomaly, unspecified |  |
| Valve | 4 | Pulmonary Valve | 746.02 | Pulmonary valve stenosis |  |
| Valve | 4 | Pulmonary Valve | 746.09 | Pulmonary valve anomaly, other |  |
| Valve | 4 | Ebstein Anomaly | 746.2 | Ebstein Anomaly |  |
| Valve | 4 | Aortic Stenosis | 746.3 | Aortic valve stenosis |  |
| Valve | 4 | Aortic Insufficiency | 746.4 | Aortic insufficiency or bicuspid/unicuspid aortic valve |  |
| Valve | 4 | Mitral Stenosis | 746.5 | Mitral stenosis or mitral valve abnormalities |  |
| Valve | 4 | Mitral Insufficiency | 746.6 | Mitral insufficiency |  |
| Valve | 4 | Other Unspec. Anomaly of Heart | 746.81 | Subaortic stenosis |  |
| Valve | 4 | Other Unspec. Anomaly of Heart | 746.83 | Infundibular or subvalvar pulmonary stenosis |  |
| Valve | 4 | Coarctation | 747.1 | Coarctation of aorta |  |
| Valve | 4 | Coarctation | 747.1 | Coarctation of aorta (preductal)(postductal) |  |
| Valve | 4 | Other Unspec. Anomaly of Aorta | 747.22 | Atresia or stenosis of aorta |  |
| Valve | 4 | Pulmonary Artery | 747.3 | Anomalies of Pulmonary artery |  |
| Valve | 4 | Pulmonary Artery | 747.31 | Pulmonary artery atresia, coarctation or hypoplasia |  |
| Valve | 4 | Pulmonary Artery | 747.39 | Anomalies of Pulmonary artery, other |  |
| Other | 5 | Personal h/o CHD | V13.65 | Preg mother with CHD |  |
| Other | 5 | Pregnant mother with CHD | 648.5x | Preg mother with CHD |  |
| Other | 5 | Absence of Atrial & Ventricular Septa | 745.7 | Cor biloculare |  |
| Other | 5 | Other Unspecified Anomaly of Heart | 746.8 | Other Specified anomalies of heart |  |
| Other | 5 | Other Unspecified Anomaly of Heart | 746.82 | cor triatrium |  |
| Other | 5 | Other Unspecified Anomaly of Heart | 746.84 | Obstructive anomalies of heart |  |
| Other | 5 | Other Unspecified Anomaly of Heart | 746.85 | Coronary artery anomaly |  |
| Other | 5 | Other Unspecified Anomaly of Heart | 746.87 | Malposition of heart or apex |  |
| Other | 5 | Other Unspecified Anomaly of Heart | 746.89 | Other specified anomaly of heart (various types) |  |
| Other | 5 | Other Unspecified Anomaly of Heart | 746.9 | Unspecified defect of heart |  |
| Other | 5 | Other Unspecified Anomaly of Aorta | 747.2 | Other anomaly of the aorta |  |
| Other | 5 | Other Unspecified Anomaly of Aorta | 747.2 | Anomalies of aorta, unspecified |  |
| Other | 5 | Other Unspecified Anomaly of Aorta | 747.21 | Anomaly of aortic arch |  |
| Other | 5 | Other Unspecified Anomaly of Aorta | 747.29 | Other anomaly of aorta |  |
| Other | 5 | Anomalies of Great Veins | 747.4 | Anomalies of great veins |  |
| Other | 5 | Anomalies of Great Veins | 747.4 | Anomalies of great veins, unspecified |  |
| Other | 5 | Anomalies of Great Veins | 747.49 | Other anomalies of great veins |  |
| Other | 5 | Other Unsp. Anomaly of circulation | 747.9 | Unspecified anomalies of circulatory system |  |


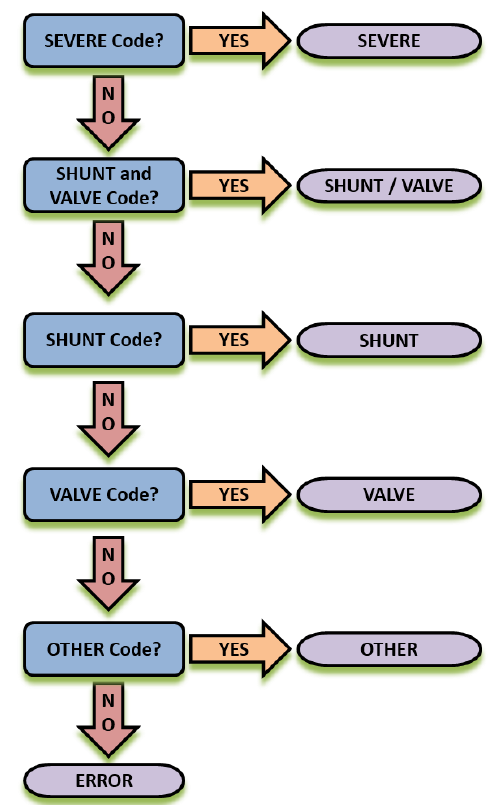
Step 2. Reconciling multiple severity groups.

For those complex cases that step 1 assigned to more than one severity group, we used the following hierarchical approach (see figure) to assign a single severity group.

Briefly, the hierarchy was severe > shunt and valve > shunt > valve > other. For example, a case with a severe code was assigned to the severe group (regardless of other group assignments).

Cases not in the severe group but with codes for the shunt and valve group were assigned to the shunt and valve group regardless of whether they had been also assigned to, for example, the ‘other CHD’ group.

Note than in this analysis the ‘other CHD’ category was excluded.

1. **Results**

Figure S1. Inclusions and exclusions leading to final study sample of adults with congenital heart disease (CHD), Lifespan study, 2011-2013


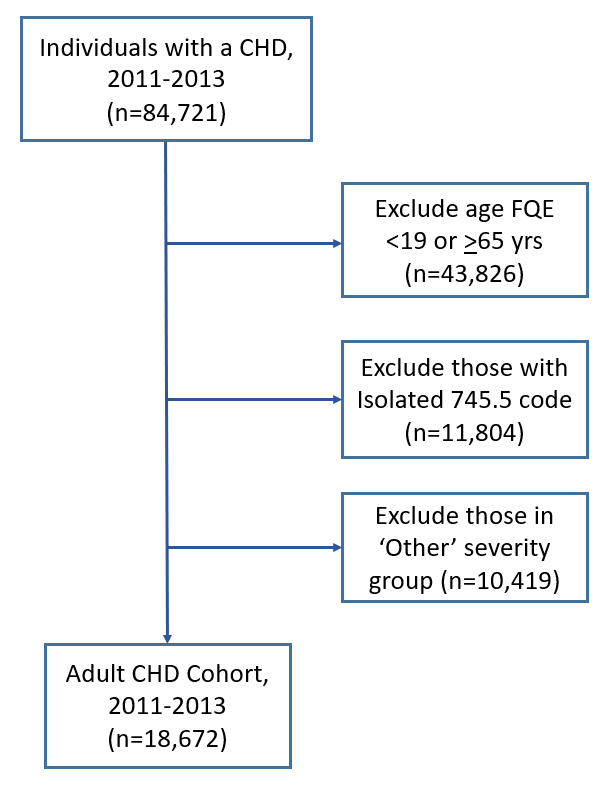


FQE, first qualifying encounter with an eligible CHD ICD-9 code in the study period (2011 through 2013)

Figure S2. Age distribution in adults 19-64 years old with congenital heart disease (CHD), by CHD group. The violin plots illustrate the density distribution by age and include boxplots with median age (dark horizontal line) and mean age (shaded diamond).


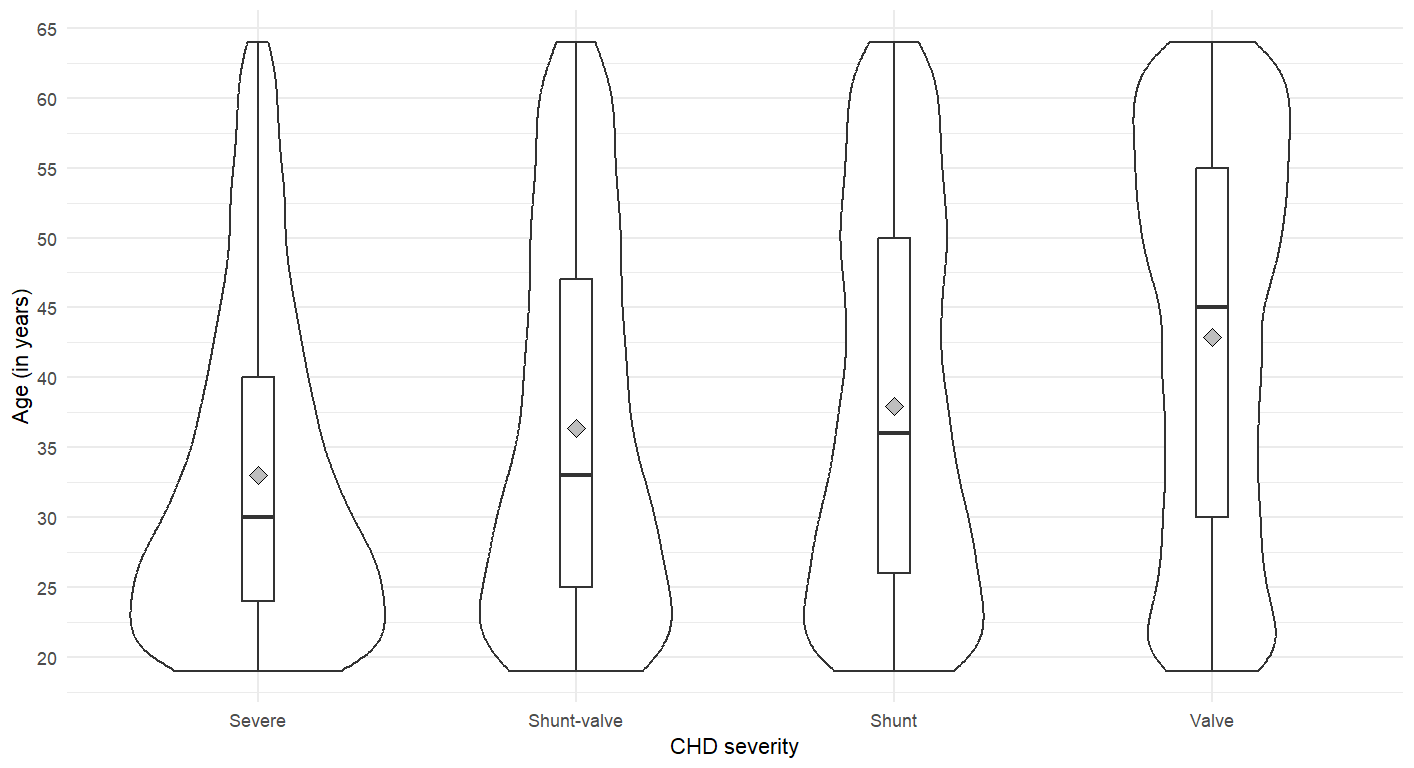


Note. All pairwise contrasts were associated with p < 0.001

**Figure S3.** Number of comorbidities by race, in the four congenital heart disease, by age group, Lifespan study, 2011-2013


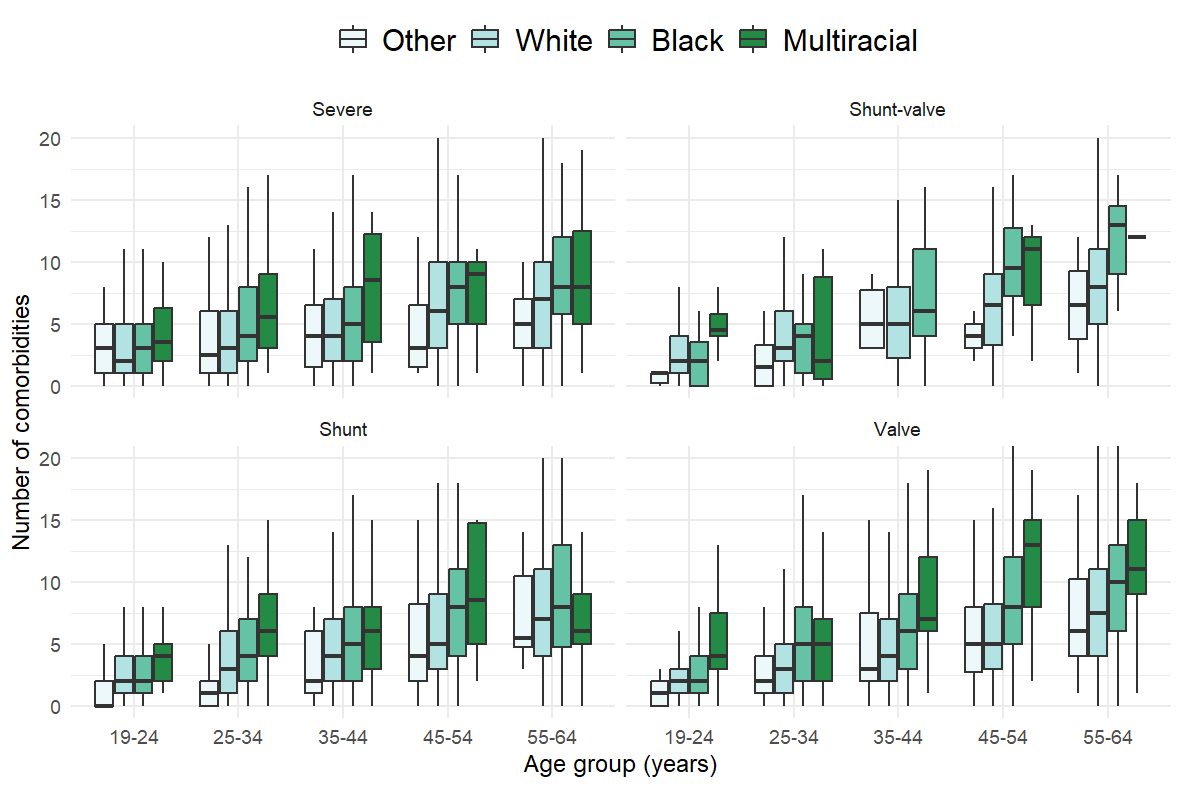


| Table S1. Major comorbidities and comorbidity patterns in adults 19-64 years old with congenital heart disease (CHD), Lifespan study, 2011-2013 | | | | | | | | | | | | | | |
| --- | --- | --- | --- | --- | --- | --- | --- | --- | --- | --- | --- | --- | --- | --- |
|  | | **Overall and by sex** | | | | **< 40 years** | | | | | **40 years and above** | | | |
| **Comorbidity** | **Overall**, N = 18,672 | | **Female**, N = 9,665 | **Male**, N = 9,003 | **Severe**,N = 2,900 | | **Shunt-valve**, N = 576 | **Shunt**, N = 2,409 | **Valve**, N = 3,898 | **Severe**, N = 1,011 | | **Shunt-valve**, N = 354 | **Shunt**, N = 1,788 | **Valve**, N = 5,736 |
| Any comorbidity, n (%) | 16,522 (89) | | 8,413 (87) | 8,109 (90) | 2,465 (85) | | 481 (84) | 1,891 (78) | 3,239 (83) | 933 (92) | | 339 (96) | 1,680 (94) | 5,498 (96) |
| Multiple comorbidities, n (%) | 14,152 (76) | | 7,132 (74) | 7,020 (78) | 1,971 (68) | | 398 (69) | 1,515 (63) | 2,502 (64) | 833 (82) | | 323 (91) | 1,545 (86) | 5,068 (88) |
| Cardiac comorbidity, n (%) | 12,469 (67) | | 5,864 (61) | 6,605 (73) | 1,896 (65) | | 374 (65) | 929 (39) | 2,359 (61) | 786 (78) | | 301 (85) | 1,162 (65) | 4,664 (81) |
| Non-cardiac comorbidity, n (%) | 15,161 (81) | | 7,656 (79) | 7,117 (79) | 2,053 (71) | | 414 (72) | 1,745 (72) | 2,670 (69) | 834 (82) | | 321 (91) | 1,632 (91) | 5,108 (89) |
| Cardiac comorbidities | | | | | | | | | | | | | | |
| Residual cardiac disease, n(%) | 9,017 (48) | | 4,140 (43) | 4,877 (54) | 1,288 (44) | | 296 (51) | 453 (19) | 1,902 (49) | 516 (51) | | 244 (69) | 545 (30) | 3,774 (66) |
| Cardiac failure, n (%) | 3,631 (19) | | 1,655 (17) | 1,976 (22) | 686 (24) | | 84 (15) | 227 (9.4) | 396 (10) | 361 (36) | | 108 (31) | 399 (22) | 1,370 (24) |
| Arrhythmia, n (%) | 5,585 (30) | | 2,590 (27) | 2,995 (33) | 1,101 (38) | | 156 (27) | 430 (18) | 699 (18) | 514 (51) | | 164 (46) | 594 (33) | 1,927 (34) |
| Coronary artery d., n (%) | 2,981 (16) | | 1,190 (12) | 1,791 (20) | 214 (7.4) | | 41 (7.1) | 155 (6.4) | 209 (5.4) | 201 (20) | | 100 (28) | 434 (24) | 1,628 (28) |
| Other cardiovascular disease, n (%) | 1,897 (10) | | 886 (9.2) | 1,011 (11) | 247 (8.5) | | 58 (10) | 151 (6.3) | 286 (7.3) | 135 (13) | | 69 (19) | 191 (11) | 760 (13) |
| Aortic, n (%) | 2,352 (13) | | 777 (8.0) | 1,575 (17) | 144 (5.0) | | 40 (6.9) | 33 (1.4) | 571 (15) | 71 (7.0) | | 63 (18) | 75 (4.2) | 1,355 (24) |
| Other vascular, n (%) | 1,899 (10) | | 961 (9.9) | 938 (10) | 194 (6.7) | | 35 (6.1) | 153 (6.4) | 196 (5.0) | 140 (14) | | 67 (19) | 294 (16) | 820 (14) |
| Non-cardiac comorbidities | | | | | | | | | | | | | | |
| Hypertension, n (%) | 6,560 (35) | | 2,894 (30) | 3,666 (41) | 398 (14) | | 97 (17) | 327 (14) | 832 (21) | 424 (42) | | 188 (53) | 939 (53) | 3,358 (59) |
| Obstructive pulmonary, n (%) | 4,147 (22) | | 2,192 (23) | 1,955 (22) | 421 (15) | | 84 (15) | 424 (18) | 566 (15) | 265 (26) | | 114 (32) | 566 (32) | 1,708 (30) |
| Pulmonary vascular, n (%) | 1,069 (5.7) | | 638 (6.6) | 431 (4.8) | 173 (6.0) | | 28 (4.9) | 125 (5.2) | 118 (3.0) | 108 (11) | | 46 (13) | 179 (10) | 292 (5.1) |
| Other pulmonary, n (%) | 5,207 (28) | | 2,600 (27) | 2,607 (29) | 704 (24) | | 134 (23) | 451 (19) | 618 (16) | 392 (39) | | 176 (50) | 657 (37) | 2,075 (36) |
| Renal, n (%) | 2,340 (13) | | 1,019 (11) | 1,321 (15) | 217 (7.5) | | 31 (5.4) | 159 (6.6) | 271 (7.0) | 177 (18) | | 57 (16) | 353 (20) | 1,075 (19) |
| Hepatic, n (%) | 2,092 (11) | | 989 (10) | 1,103 (12) | 317 (11) | | 38 (6.6) | 146 (6.1) | 253 (6.5) | 166 (16) | | 53 (15) | 292 (16) | 827 (14) |
| Gastrointestinal, n (%) | 4,957 (27) | | 2,652 (27) | 2,305 (26) | 491 (17) | | 91 (16) | 530 (22) | 673 (17) | 344 (34) | | 131 (37) | 654 (37) | 2,043 (36) |
| Neurologic, n (%) | 3,451 (18) | | 1,731 (18) | 1,720 (19) | 402 (14) | | 84 (15) | 362 (15) | 418 (11) | 252 (25) | | 111 (31) | 529 (30) | 1,293 (23) |
| Hematologic, n (%) | 5,432 (29) | | 2,830 (29) | 2,602 (29) | 661 (23) | | 123 (21) | 579 (24) | 683 (18) | 392 (39) | | 170 (48) | 642 (36) | 2,182 (38) |
| Nutrition, n (%) | 3,559 (19) | | 2,083 (22) | 1,476 (16) | 400 (14) | | 95 (16) | 450 (19) | 478 (12) | 209 (21) | | 95 (27) | 460 (26) | 1,372 (24) |
| Orthopedic, n (%) | 2,213 (12) | | 1,266 (13) | 947 (11) | 153 (5.3) | | 33 (5.7) | 127 (5.3) | 203 (5.2) | 163 (16) | | 60 (17) | 383 (21) | 1,091 (19) |
| Endocrine, n (%) | 6,985 (37) | | 3,438 (36) | 3,547 (39) | 484 (17) | | 108 (19) | 515 (21) | 707 (18) | 470 (46) | | 192 (54) | 1,055 (59) | 3,457 (60) |
| Immunologic, n (%) | 599 (3.2) | | 418 (4.3) | 181 (2.0) | 74 (2.6) | | 8 (1.4) | 55 (2.3) | 80 (2.1) | 38 (3.8) | | 19 (5.4) | 80 (4.5) | 245 (4.3) |
| Learning or intellectual, n (%) | 484 (2.6) | | 203 (2.1) | 281 (3.1) | 100 (3.4) | | 28 (4.9) | 86 (3.6) | 106 (2.7) | 26 (2.6) | | 8 (2.3) | 45 (2.5) | 85 (1.5) |
| Psychiatric, n (%) | 7,155 (38) | | 3,621 (37) | 3,534 (39) | 828 (29) | | 182 (32) | 821 (34) | 1,191 (31) | 413 (41) | | 169 (48) | 864 (48) | 2,688 (47) |
| Genetic syndromes, n (%) | 807 (4.3) | | 411 (4.3) | 396 (4.4) | 285 (9.8) | | 40 (6.9) | 180 (7.5) | 92 (2.4) | 56 (5.5) | | 11 (3.1) | 81 (4.5) | 62 (1.1) |
| Oncologic, n (%) | 3,080 (16) | | 1,729 (18) | 1,351 (15) | 243 (8.4) | | 46 (8.0) | 248 (10) | 348 (8.9) | 205 (20) | | 78 (22) | 459 (26) | 1,453 (25) |

Table S2. Summary of current study and prior studies 2018-2023.

| Reference | Time, Place, Data Source(s) | Study Population | Main Findings | Notes / Details |
| --- | --- | --- | --- | --- |
| This study, Botto et al., 2024 | - 2011-2013 - US (Atlanta, Georgia; Colorado, New York State (partial), North Carolina, Utah ) - Linked administrative and clinical databases | N = 18 672 with CHD  19 to 64 years of age  Retrospective cohort  21% with severe CHD | with at least on comorbidity: 89%  with multimorbidity (> 1 comorbidity): 76%  noncardiac (81%) > cardiac (62%)  positively associated with age, public insurance, and Black/multiracial race  most common:   - endocrine and metabolic (37%) - hypertensions (35%) - psychiatric (38%) | - population-based, five geographic areas in US - inpatient, outpatient, emergency room encounters - excludes isolated atrial septal defect, patent foramen ovale, and other minor conditions |
| Maurer et al., 2021 | - 2012-17 - Germany - National CHD Registry | N = 4 673 with CHD  >18 years of age  Cross sectional study | Comorbidities by age and type   - at least one: 61.7%   <40 years = 56.8%  >40 years = 77.7%   - average number per patient:   <40 years =1.2+/-1.5  >40 years =2.1+/-2.1   - most common: - endocrine and metabolic (30.4%) - circulatory system disorders (28.2%) - nervous system (11.5%) - positively associated with age and CHD severity | - National CHD registry |
| Oster et al., 2021 | - 2016-2019 - US (Arizona, Arkansas, Georgia) - Self-report survey - Individuals identified through birth defect registries | N = 1 482 with CHD  20-38 years of age  33% with severe CHD  Survey respondents (24% response rate)  Compared with published US population data | Includes cardiac and non-cardiac comorbidities;  compared severe vs. less severe CHD (table 2 of report)   - at least one comorbidity ~14% (estimated from figure, data not provided) - Frequency of selected comorbidities - depression (15.1%) - stroke (1.4%) - more common in severe vs. less severe CHD: - at least 1 cardiac: aOR=2.4 (95% CI 1.7-3.3) - congestive heart failure: aOR=4.4 (2.5-7.5) - hypertension: aOR=1.9 (1.3-2.8) - stroke: aOR=3.8, (1.5-9.7) - No difference in non-cardiac comorbidities between severe and non-severe CHD | - Survey based (self- reported or by proxy) - Young adults only - 24% survey response rate - Survey used questions from two large population survey (NHANES, BRFSS), allowing comparisons with population data |
| Gurvitz et al., 2020 | - 2008-2010 - US (Atlanta, Georgia; Massachusetts; New York State 11 counties) - CHD-coded encounters in outpatient and inpatient administrative databases | N = not stated  20–64-year-olds  20% with severe CHD  Cross sectional study | Compared severe vs. non severe CHD   - at least one cardiac comorbidity: >50% - most common cardiac comorbidities: - conduction and/or rhythm disorder (26-50%) - stroke and/or thrombosis or other cardiovascular conditions (5-75%) - most common noncardiac comorb: - respiratory and/or pulmonary (33-51%) - gastrointestinal (21-46%) - infectious disease (21-38%). | - Includes residents during 2008-2010 with eligible health encounter with CHD code. - Excludes isolated atrial septal defect or patent foramen ovale |
| Agarwal et al., 2019 | - 2010-2016 - United States (partial) - IBM Marketscan Claims Data - inclusive of inpatient & outpatient claims data on IBM employees and families with > 2 years of continuous employment | N = 40 127 with CHD  18 to 64 years of age  Included matched controls without CHD  Cross sectional study | Compared people with CHD vs. without CHD, using adjusted rate ratios (aRR)   - Comorbidities at age 18-40 years - any: aRR = 1.16 (1.1-1.2) - common in CHD: aRR=3.3 (2.7-3.9) - other cardiac: aRR = 0.84 (0.74-0.96) - noncardiac: aRR = 1.19 (1.09-1.29) - Comorbidities at age > 40 years - any: aRR= 1.04 (0.99-1.08) - common in CHD: aRR =1.96 (1.76-2.19) - other cardiac: aRR = 0.91 (0.86-0.96) - noncardiac: aRR = 1.00 (0.94-1.07) | - Based on administrative (health insurance) data set - Excluded claims from Medicare, Medicaid, and Workers Comp - Selected population (employees/families participating in one specific health insurance system) |
| Neidenbach et al., 2018 | - Published 2018 - Germany, Munich - Single tertiary care center | N = 821  >18 years (median 34.8)  Cross sectional study | Focus on non-cardiac comorbidities   - at least one: 95.2% - most common: - endocrine and metabolic (41.9%) - gastroenterology/liver (30.8%) - musculoskeletal (28.4%) - neurologic (18.0%) - hypertension (16.4%) - pulmonary (15.3%) | - Based on medical record review - Included genetic syndromes (11.4%) - Single tertiary care center - Unclear study period |
| Singh et al., 2018 | - 2013-2014 - US (sample) - National Inpatient Sample (NIS) Database | N = 255 355 hospitalizations  Adults (lower age limit not given)  Mean age at hospitalization, 56.9 years | Focus on noncardiac comorbidities   - at least one: data not provided - most common - Hypertension (54.9%) - Dyslipidemia (39.6%) - Fluid/electrolyte disturbance (27.9%) - Chronic obstructive pulm. disease (20.4%) - Diabetes mellitus (17.8%) - Anemia (16.8%) - Obesity (14.8%) | - Inpatient only - Nationally representative - Deidentified sample - Based on ~20% of community hospitals in US - Includes all discharges regardless of expected payer status |

1. Missing data analysis.

Among the covariates included in the analysis, three (race, ethnicity, and insurance type) had >1% missing data (table)

| Variable | N missing | % |
| --- | --- | --- |
| Race | 3457 | 18.5 |
| Ethnicity | 3241 | 17.4 |
| Insurance | 1076 | 5.76 |

The pattern of missingness among the three covariates is shown in the figure below.


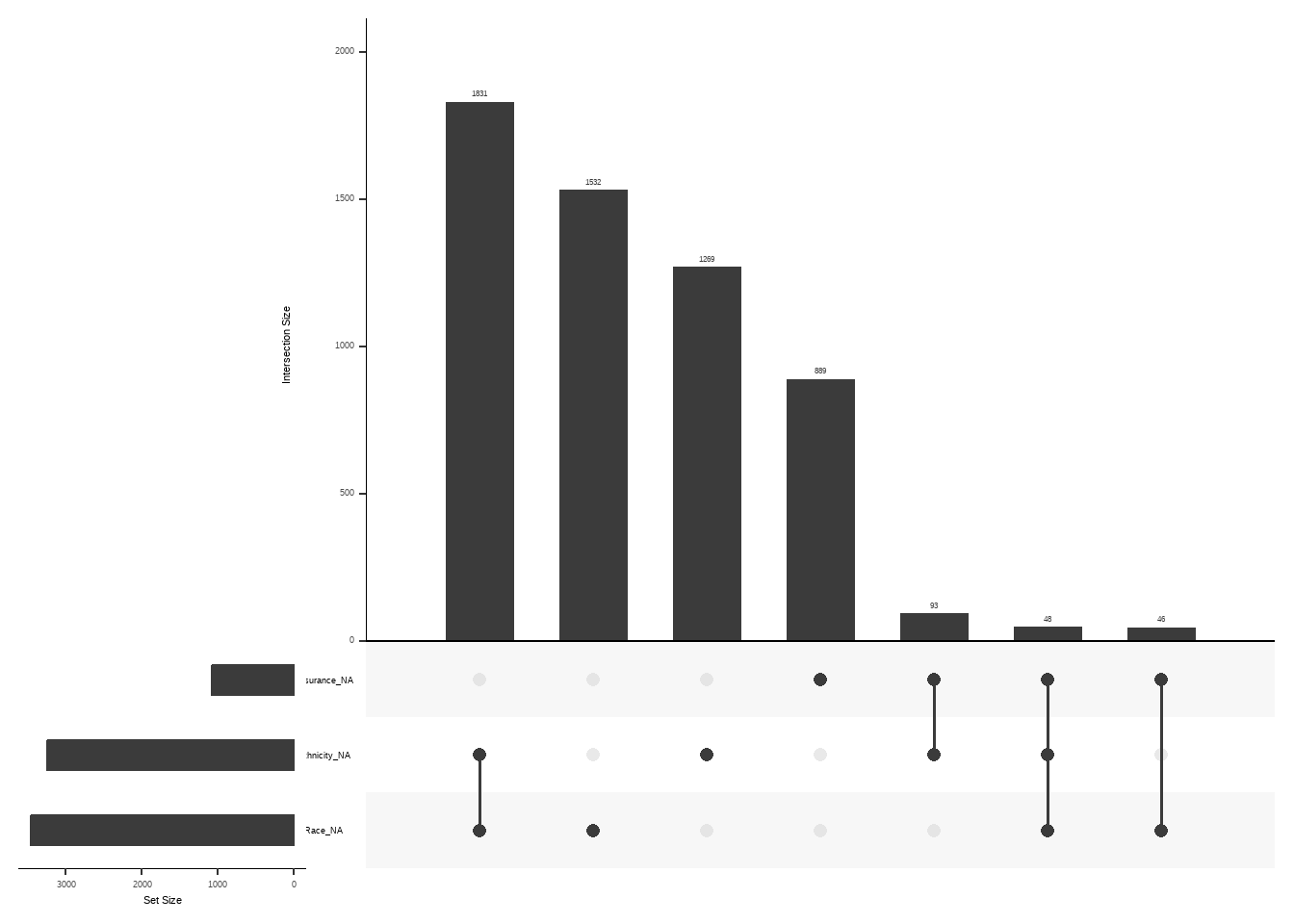


The correlation between missingness among the three variables was modest. Missingness in ethnicity was modestly correlated to insurance type but not with race.

To assess the effect of missingness on study findings we used several multivariable imputation models, including random forest (single imputation, using the missRanger package in R) and multiple imputation by chained equation (MICE), using the MICE package in R and replicated in SAS software package.

MICE is a robust statistical technique that operates under the assumption that the data are missing at random (MAR), meaning the propensity for missingness is related to observed data but not the missing data itself. MICE fills in the missing values multiple times to create several complete datasets, which allows for more accurate statistical analysis by addressing the uncertainty associated with missing data (see references below).

The process begins by initializing missing values with mean or median estimates. Then, for each incomplete variable, a regression model is built using the other variables in the dataset. MICE iteratively cycles through each variable, predicting the missing values based on the observed values of other variables, and updates the imputed values.

This procedure is repeated several times (m iterations) to stabilize the imputed values. The multiple datasets created are analyzed separately, and the results are combined using Rubin's rules to produce final parameter estimates and standard errors that reflect the variability between imputations.

MICE is particularly advantageous because it can handle different types of variables (continuous, binary, categorical) and complex datasets with various missing data patterns. It also allows for the incorporation of auxiliary variables that may not be part of the final analysis but help in the imputation process.

Because MICE is generally considered superior to single imputation (as was used in the random forest model), details and findings using MICE are here summarized.

In the implementation of MICE (‘mice’ R package) for this study, the prediction matrix included as predictors age (in years), birth year, study site, CHD type, and Charlson score. Sex, race, ethnicity, and insurance type were the predicted variables.

The procedure was run for m (number of imputed datasets) equal to 5, 10, and 20 and pooled effect estimates (incidence risk ratios) were then computed. The findings using these different numbers of dataset were virtually identical. The procedure was implemented independently in R and SAS software.

Shown below is Table 2 comparing the original estimates with the those using MICE, with m (number of imputed datasets) equal to five. Inspection of the table shows that the two sets of estimates are very similar. For this reason, we elected to keep to original analysis in the main report.

| **Any comorbidity** | **Original Values** | | | | **MICE Imputation** | | |
| --- | --- | --- | --- | --- | --- | --- | --- |
| **Characteristic** |  | **IRR** **(95% CI)***^1^* | **p-value** | **q-value***^2^* | **IRR** **(95% CI)***^1^* | **p-value** | **q-value***^2^* |
| **Age group, n (%)** |  |  | <0.001 | <0.001 |  |  |  |
| 19-24 |  | 1.00 |  |  | 1.00 |  |  |
| 25-34 |  | 1.06 (1.04 to 1.09) | <0.001 | <0.001 | 1.08 (1.07 to 1.11) | <0.001 | <0.001 |
| 35-44 |  | 1.13 (1.10 to 1.15) | <0.001 | <0.001 | 1.17 (1.15 to 1.20) | <0.001 | <0.001 |
| 45-54 |  | 1.17 (1.15 to 1.20) | <0.001 | <0.001 | 1.23 (1.21 to 1.25) | <0.001 | <0.001 |
| 55-64 |  | 1.18 (1.16 to 1.21) | <0.001 | <0.001 | 1.25 (1.23 to 1.27) | <0.001 | <0.001 |
| **Sex, n (%)** |  |  | 0.53 | 0.58 |  |  |  |
| Female |  | 1.00 |  |  | 1.00 |  |  |
| Male |  | 1.01 (1.00 to 1.02) | 0.019 | 0.032 | 1.02 (1.02 to 1.04) | <0.001 | <0.001 |
| Unknown |  |  |  |  |  |  |  |
| **Race, n (%)** |  |  | 0.026 | 0.038 |  |  |  |
| White |  | 1.00 |  |  | 1.00 |  |  |
| Black |  | 1.03 (1.02 to 1.04) | <0.001 | <0.001 | 1.02 (1.00 to 1.04) | 0.043 | 0.051 |
| Other |  | 0.87 (0.83 to 0.91) | <0.001 | <0.001 | 0.86 (0.81 to 0.90) | <0.001 | <0.001 |
| Multiracial |  | 1.05 (1.03 to 1.07) | <0.001 | <0.001 | 1.05 (1.00 to 1.10) | 0.042 | 0.051 |
| Unknown |  |  |  |  |  |  |  |
| **Ethnicity, n (%)** |  |  | 0.57 | 0.59 |  |  |  |
| non-Hispanic |  | 1.00 |  |  | 1.00 |  |  |
| Hispanic |  | 1.02 (1.00 to 1.04) | 0.023 | 0.035 | 1.03 (1.00 to 1.04) | 0.007 | 0.010 |
| Unknown |  |  |  |  |  |  |  |
| **Insurance type, n (%)** |  |  | <0.001 | <0.001 |  |  |  |
| Public |  | 1.00 |  |  | 1.00 |  |  |
| Private |  | 0.92 (0.91 to 0.93) | <0.001 | <0.001 | 0.91 (0.90 to 0.92) | <0.001 | <0.001 |
| Other |  | 0.92 (0.88 to 0.96) | <0.001 | <0.001 | 0.92 (0.88 to 0.96) | <0.001 | <0.001 |
| Unknown |  |  |  |  |  |  |  |
| **Site, n (%)** |  |  | 0.028 | 0.038 |  |  |  |
| North Carolina |  | 1.00 |  |  | 1.00 |  |  |
| Colorado |  | 0.95 (0.93 to 0.96) | <0.001 | <0.001 | 0.94 (0.93 to 0.96) | <0.001 | <0.001 |
| New York |  | 0.94 (0.93 to 0.95) | <0.001 | <0.001 | 1.00 (0.99 to 1.02) | 0.951 | 0.951 |
| Utah |  | 1.01 (1.00 to 1.03) | 0.049 | 0.062 | 1.08 (1.07 to 1.10) | <0.001 | <0.001 |
| Georgia |  | 0.94 (0.92 to 0.96) | <0.001 | <0.001 | 0.97 (0.96 to 1.00) | 0.04 | 0.050 |
| **Severity class, n (%)** |  |  | 0.78 | 0.78 |  |  |  |
| Severe |  | 1.00 |  |  | 1.00 |  |  |
| Shunt-valve |  | 1.01 (0.99 to 1.04) | 0.28 | 0.33 | 1.00 (0.98 to 1.01) | 0.665 | 0.748 |
| Shunt |  | 0.98 (0.96 to 1.00) | 0.032 | 0.043 | 0.96 (0.94 to 0.97) | <0.001 | <0.001 |
| Valve |  | 1.00 (0.99 to 1.02) | 0.53 | 0.58 | 1.00 (0.98 to 1.03) | 0.73 | 0.775 |
|  |  |  |  |  |  |  |  |
| **Number of comorbidities** | **Original Values** | | | | **SAS Mice values** | | |
| **Characteristic** |  | **IRR** **(95% CI)***^1^* | **p-value** | **q-value***^2^* | **IRR** **(95% CI)***^1^* | **p-value** | **q-value***^2^* |
| **Age group, n (%)** |  |  | <0.001 | <0.001 |  |  |  |
| 19-24 |  | 1.00 |  |  | 1.00 |  |  |
| 25-34 |  | 1.33 (1.28 to 1.39) | <0.001 | <0.001 | 1.36 (1.30 to 1.42) | <0.001 | <0.001 |
| 35-44 |  | 1.75 (1.67 to 1.84) | <0.001 | <0.001 | 1.83 (1.76 to 1.91) | <0.001 | <0.001 |
| 45-54 |  | 2.26 (2.17 to 2.36) | <0.001 | <0.001 | 2.39 (2.30 to 2.48) | <0.001 | <0.001 |
| 55-64 |  | 2.63 (2.52 to 2.75) | <0.001 | <0.001 | 2.80 (2.70 to 2.91) | <0.001 | <0.001 |
| **Sex, n (%)** |  |  | <0.001 | <0.001 |  |  |  |
| Female |  | 1.00 |  |  | 1.00 |  |  |
| Male |  | 1.07 (1.04 to 1.10) | <0.001 | <0.001 | 1.08 (1.06 to 1.10) | <0.001 | <0.001 |
| Unknown |  |  |  |  |  |  |  |
| **Race, n (%)** |  |  | <0.001 | <0.001 |  |  |  |
| White |  | 1.00 |  |  | 1.00 |  |  |
| Black |  | 1.07 (1.04 to 1.11) | <0.001 | <0.001 | 1.04 (1.01 to 1.08) | 0.015 | 0.018 |
| Other |  | 0.70 (0.64 to 0.76) | <0.001 | <0.001 | 0.70 (0.63 to 0.77) | <0.001 | <0.001 |
| Multiracial |  | 1.23 (1.16 to 1.30) | <0.001 | <0.001 | 1.24 (1.17 to 1.33) | <0.001 | <0.001 |
| Unknown |  |  |  |  |  |  |  |
| **Ethnicity, n (%)** |  |  | <0.001 | <0.001 |  |  |  |
| non-Hispanic |  | 1.00 |  |  | 1.00 |  |  |
| Hispanic |  | 1.08 (1.03 to 1.13) | <0.001 | <0.001 | 1.03 (1.00 to 1.07) | 0.041 | 0.046 |
| Unknown |  |  |  |  |  |  |  |
| **Insurance type, n (%)** |  |  | <0.001 | <0.001 |  |  |  |
| Public |  | 1.00 |  |  | 1.00 |  |  |
| Private |  | 0.58 (0.56 to 0.59) | <0.001 | <0.001 | 0.57 (0.56 to 0.58) | <0.001 | <0.001 |
| Other |  | 0.57 (0.52 to 0.63) | <0.001 | <0.001 | 0.58 (0.53 to 0.63) | <0.001 | <0.001 |
| Unknown |  |  |  |  |  |  |  |
| **Site, n (%)** |  |  | <0.001 | <0.001 |  |  |  |
| North Carolina |  | 1.00 |  |  |  |  |  |
| Colorado |  | 0.86 (0.83 to 0.90) | <0.001 | <0.001 | 0.86 (0.83 to 0.89) | <0.001 | <0.001 |
| New York |  | 0.87 (0.84 to 0.90) | <0.001 | <0.001 | 1.02 (0.99 to 1.05) | 0.260 | 0.275 |
| Utah |  | 1.08 (1.04 to 1.13) | <0.001 | <0.001 | 1.23 (1.19 to 1.27) | <0.001 | <0.001 |
| 11 Georgia |  | 0.94 (0.90 to 0.98) | 0.009 | 0.010 | 1.06 (1.01 to 1.10) | 0.009 | 0.012 |
| **Severity class, n (%)** |  |  | <0.001 | <0.001 |  |  |  |
| Severe |  | 1.00 |  |  |  |  |  |
| Shunt-valve |  | 1.07 (1.01 to 1.13) | 0.037 | 0.038 | 1.09 (1.03 to 1.15) | 0.002 | 0.002 |
| Shunt |  | 0.93 (0.89 to 0.96) | <0.001 | <0.001 | 0.91 (0.88 to 0.95) | <0.001 | <0.001 |
| Valve |  | 0.99 (0.96 to 1.02) | 0.28 | 0.28 | 0.99 (0.96 to 1.03) | 0.739 | 0.739 |

References:

Rubin, D. B. (1987). Multiple Imputation for Nonresponse in Surveys. John Wiley & Sons.

van Buuren, S., & Groothuis-Oudshoorn, K. (2011). MICE: Multivariate imputation by chained equations in R. Journal of Statistical Software, 45(3), 1-67.

White, I. R., Royston, P., & Wood, A. M. (2011). Multiple imputation using chained equations: Issues and guidance for practice. Statistics in Medicine, 30(4), 377-399.

‘mice’ package documentation: <https://cran.r-project.org/web/packages/mice/mice.pdf> (updated June 2023)
